# A Method for Determining the Moisture Content of Sand Using Infrared Imaging Spectroscopy

**DOI:** 10.3390/s26216857

**Published:** 2026-10-29

**Authors:** Tomasz Sosnowski, Klaudia Majchrowicz

**Affiliations:** Institute of Optoelectronics, Military University of Technology, 00-908 Warsaw, Poland; klaudia.majchrowicz@wat.edu.pl

**Keywords:** non-contact sand moisture measurement, infrared imaging spectroscopy, infrared hyperspectroscopy, signal processing

## Abstract

A method for determining the moisture content of sand based on hyperspectral imaging in the near-infrared (NIR) spectral range is presented. The proposed approach provides a non-contact means of estimating material moisture content and has the potential for future application in large-scale moisture monitoring systems. A model describing the relationship between the moisture content of sand and the spectral signal recorded by a hyperspectral camera was developed. The derivation of the model equations and the justification of the adopted assumptions are presented. Based on an analysis of the physical phenomena occurring during the interaction of electromagnetic radiation with moist sand, a mathematical function describing the relationship between moisture content and the recorded spectral signal was proposed. The algorithm implementing the proposed method was developed in Python and enabled hyperspectral data processing, model parameterization using the acquired datasets, and validation of the approximation results. The proposed method was experimentally evaluated using data acquired under controlled laboratory conditions with a Specim FX17 hyperspectral camera. The study demonstrates the possibility of using near-infrared hyperspectral data to estimate sand moisture content and provides a basis for further research into non-contact moisture monitoring. With further development and validation, the method may find applications in environmental monitoring, precision agriculture, and airborne and satellite hyperspectral remote sensing.

## 1. Introduction

Technological advances in infrared and thermal imaging have led to the widespread application of thermal imaging and hyperspectral cameras operating in the infrared spectral range. These imaging systems are extensively used in object detection systems [1], methane detection [2,3], environmental monitoring [4,5], military, police, and firefighting equipment, aviation safety and airport security, medicine [6], and scientific research, as well as in the automotive industry and industrial production monitoring systems.

Hyperspectral imaging technology provides the ability to acquire data in numerous narrow spectral bands and enables the determination of the physical and chemical properties of objects based on their spectral characteristics. Compared with conventional measurement methods, hyperspectral imaging provides a non-contact and non-destructive approach for analyzing object properties [1].

Water is a key factor determining the physical, chemical, and biological properties of soils and bulk materials such as sand. The moisture content of these materials influences their mechanical behavior, thermal conductivity, optical properties, and gas exchange with the atmosphere. In the context of precision agriculture, environmental monitoring, and remote sensing, the ability to rapidly and non-destructively estimate the spatial distribution of soil moisture is of fundamental importance for irrigation optimization, crop yield prediction, and hydrological process modeling.

Conventional methods for determining soil moisture content, such as the gravimetric method, require collecting soil samples in the field and drying them under laboratory conditions. This process is time-consuming and prevents continuous and rapid monitoring over large areas. Alternative approaches, including capacitance sensors, resistive sensors, and neutron-based methods, enable in situ measurements but are characterized by limited spatial coverage and often require calibration for specific soil types. Advances in remote sensing technologies [1], particularly imaging spectroscopy in the near-infrared (NIR) spectral range, have opened new possibilities for the non-contact assessment of material moisture based on their spectral signatures.

Near-infrared (NIR) spectroscopy (700–2500 nm) is based on the fact that water exhibits characteristic absorption bands in this spectral region, resulting from molecular vibrations [7]. The water absorption bands in the NIR region, located around wavelengths of 970 nm, 1160 nm, 1440 nm, and 1930 nm, vary in depth and shape depending on the water content of the material. This relationship provides the physical basis for the development of quantitative models that enable the conversion of the recorded spectral signal into the moisture content of the investigated material. In the case of sand, which is characterized by a high quartz content and a relatively homogeneous grain structure, the spectral signal in the NIR range is particularly sensitive to changes in moisture content, making sand a suitable material for testing and validating new measurement methods.

Remote sensing methods based on hyperspectral data enable the estimation of moisture content not only at individual measurement points but also over large areas with spatial continuity, which is not possible using conventional measurement techniques. Hyperspectral cameras, such as the Specim FX17, acquire images in hundreds of narrow spectral bands, providing complete reflectance and radiance spectra for each pixel of the observed scene. As a result, it is possible not only to estimate the average moisture content of a given area but also to analyze its spatial variability, which is essential in applications such as precision agriculture, drought monitoring, and airborne and satellite remote sensing.

This paper presents a method developed for determining the moisture content of sand using imaging spectroscopy in the near-infrared (NIR) spectral range. The proposed method represents an innovative approach to the non-contact determination of material moisture and has the potential to be applied in future systems for rapid large-area moisture monitoring. A model describing the relationship between sand moisture content and the spectral signal recorded by a hyperspectral camera was developed. The paper presents the derivation of the proposed model and justifies the adopted assumptions based on an analysis of the physical phenomena occurring during the interaction of electromagnetic radiation with moist sand.

## 2. Measurement System

The near-infrared hyperspectral imaging (NIR-HSI) measurement system is designed to simultaneously acquire spatial and spectral information, enabling the determination of reflection, transmission, or reflectance spectra for each pixel of the image [8]. In practical measurement applications, the near-infrared spectral range typically covers wavelengths from 900 to 1700 nm, allowing the detection of characteristic absorption bands of water, organic matter, and minerals in soil, vegetation, and environmental materials [8,9].

In laboratory applications, NIR-HSI systems are widely used for the analysis of soil, agricultural, and technical materials. It enables the assessment of material homogeneity, the generation of physicochemical parameter maps, and the analysis of changes in moisture content or chemical composition as a function of position within the sample.

**Figure 1:**
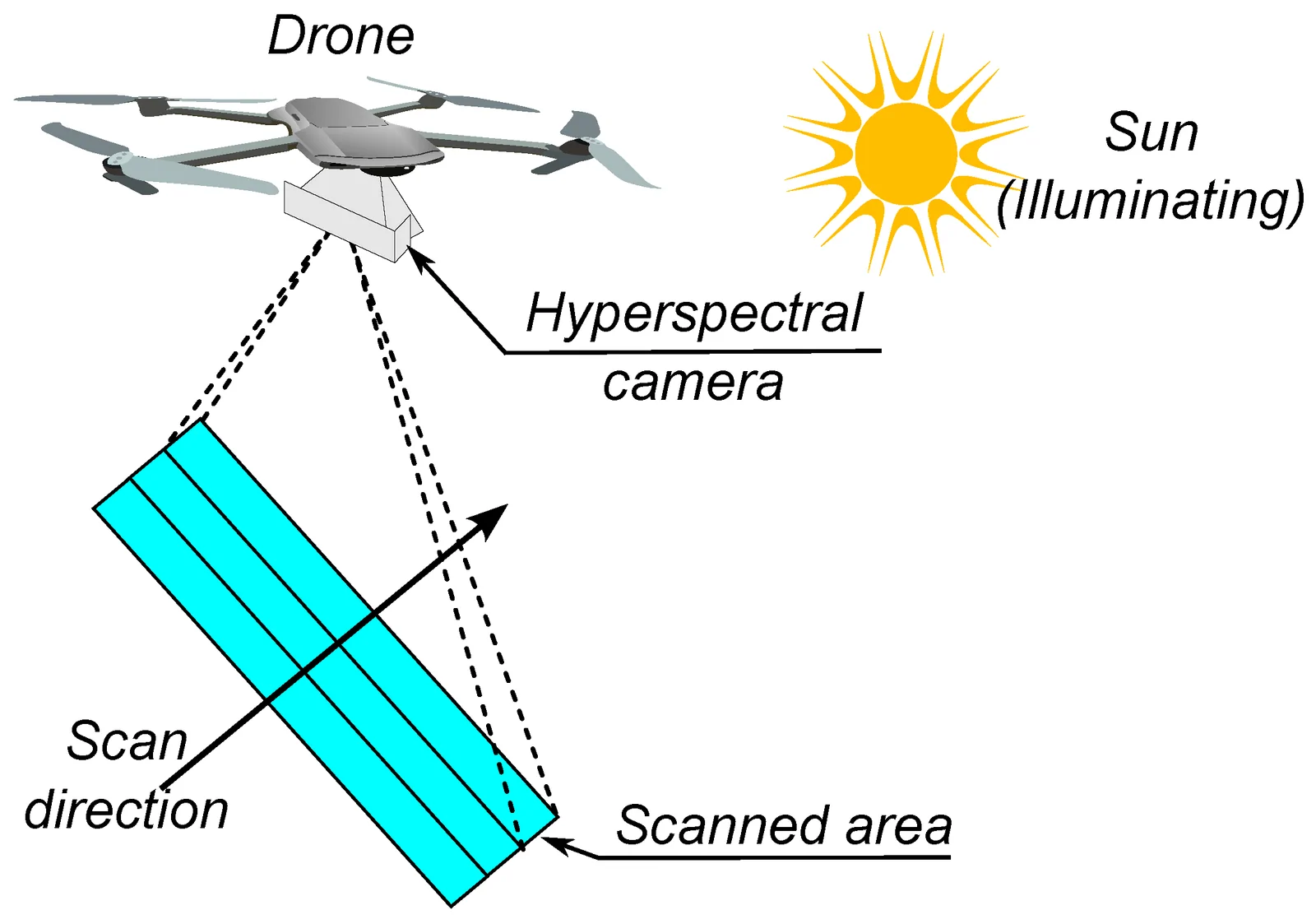
Schematic diagram of a field hyperspectral image acquisition system using a drone-mounted camera.

The NIR-HSI measurement system consists of the following main components: an NIR radiation source (for the observed object or scene), a sample illumination system (a laboratory lamp or sunlight for field measurements), a hyperspectral camera with an objective, a sample positioning system, a data acquisition system, and a control system [10,11]. A schematic diagram of the measurement system is shown in Figure 1.

In laboratory environments, NIR illumination is typically provided by high-power halogen lamps or broadband LED light sources, which ensure uniform illumination of the investigated sample [8]. The illumination system includes optical homogenizing components, such as collimators and diffusing lenses, whose purpose is to provide a uniform radiation intensity across the entire field of view of the hyperspectral camera. For field measurements, the Sun serves as the illumination source, and the illumination conditions vary both spatially and temporally. Therefore, for systems intended to operate in outdoor environments, compensation for illumination variations should be considered already at the stage of developing data processing methods and designing the measurement system.

The key component of an NIR-HSI system is the imaging spectrometer, which transforms the image of the sample into a hyperspectral data cube. In laboratory and remote sensing applications, pushbroom imaging systems are the most widely used. In these systems, the camera acquires the image of a single line of the sample, while the spectrum is dispersed in the direction perpendicular to the line [9,10]. Each pixel of the acquired line contains a complete spectrum within the operating spectral range (e.g., 900–1700 nm), and the movement of the sample relative to the spectrometer entrance slit enables the reconstruction of the second spatial dimension. A pushbroom imaging system consists of:

- An objective that images the sample onto the entrance slit;
- An entrance slit defining the spatial resolution along the scanned line;
- A collimator forming a parallel light beam;
- A diffraction element (diffraction grating) that disperses the spectrum;
- An imaging lens that projects the dispersed spectrum onto the detector array.

**Figure 2:**
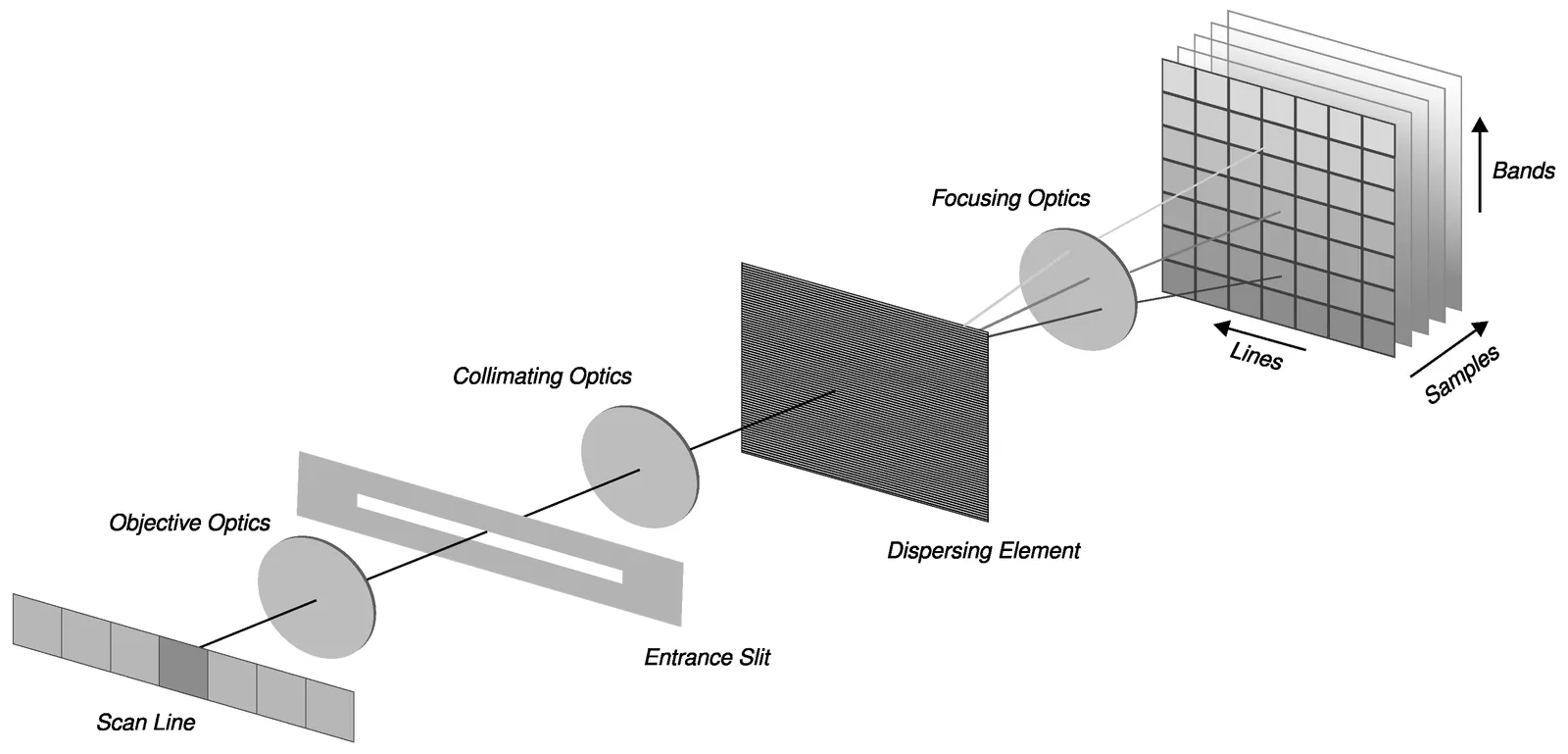
Simplified schematic diagram of the hyperspectral camera.

A simplified schematic of the hyperspectral camera is shown in Figure 2.

An NIR hyperspectral camera incorporates a detector array (typically based on InGaAs technology for the 900–1700 nm spectral range) together with signal readout and processing electronics [8,12]. The detector array is selected to provide an appropriate number of spatial pixels and spectral channels (typically several hundred spectral bands) [10]. The key performance parameters of the camera include:

- Quantum efficiency in the NIR spectral range;
- Detector readout noise and dark current;
- Maximum line acquisition rate (for pushbroom systems);
- Analog-to-digital converter (ADC) resolution (typically 14–16 bits).

Comparative analyses of different detector configurations and signal readout architectures have demonstrated the influence of these parameters on spectral quality and on the capability of acquiring hyperspectral data under both laboratory and field conditions [12].

In laboratory systems for the analysis of soils or solid materials, a linear translation stage or an XY translation stage is commonly used to enable precise positioning of the sample relative to the spectrometer entrance slit [11]. The movement of the sample along the axis perpendicular to the slit allows the reconstruction of the complete hyperspectral data cube (x,y,λ) from consecutively acquired image lines. Synchronization of the translation stage movement with line acquisition is essential to ensure the correct spatial representation of the data cube. Studies on pushbroom hyperspectral microscopy emphasize the importance of mechanical precision and scanning speed control for achieving high quality of spectral data [11].

For airborne systems, the pushbroom spectrometer acquires a single image line perpendicular to the flight direction (cross-track direction) at each instant. The complete hyperspectral data cube (x,y,λ) is reconstructed by sequentially combining consecutive image lines as the drone moves over the observed surface.

## 3. Measurement System Model

### 3.1. Definition of Sand Moisture Content

Soil in its natural state consists of solid particles and void spaces between them. The void spaces may be filled with air, water, or both. Therefore, soil is considered a three-phase system comprising the following phases [13]:

- Dry soil—the void spaces contain no water;
- Saturated soil—the void spaces are completely filled with water;
- Moist soil—the void spaces are partially filled with water.

When analyzing soil conditions, it is important to establish the relationship between the mass and the volume of water contained within a given mass (or volume) of soil. Water content (also referred to as moisture content) is defined as the amount of water contained in a material such as soil, sand, rock, ceramics, or wood. Moisture content can be expressed using either mass-based or volumetric parameters [13,14]. In general, soils exhibit considerable variability, and their properties depend on numerous, often complex, factors, making it difficult to perform repeatable measurements. Therefore, sand was selected for the subsequent analysis of moisture determination in granular soils, as it provides a more homogeneous material and enables repeatable measurements. Sand is a granular material consisting of solid particles and intergranular voids filled with air and water. Therefore, the description of sand moisture should take into account not only the mass of water contained in the material but also the pore volume and the degree of pore saturation. The total volume of the sample can be expressed as [13,14]:

(1) $$V_{t}=V_{d}+V_{w}+V_{a}=V_{d}+V_{p}$$

where: $V_{d}$ is the volume of the solid material (sand particles), $V_{a}$ is the volume of air, $V_{w}$ is the volume of water contained in the pores, $V_{t}$ is the total sample volume, and $V_{p}=V_{w}+V_{a}$ is the pore volume occupied by water and air.

One of the fundamental parameters used to describe the water content of a material is the degree of water saturation $S_{r}$. It is defined as the ratio of the volume of water contained in the pores to the total pore volume [13,14]:

(2) $$S_{r}=\frac{V_{w}}{V_{p}}$$

where: $V_{w}$ is the volume of water contained in the pores, and $V_{p}$ is the total pore volume.

An important parameter used to characterize porous materials such as sand is the porosity, *n*, which describes the ratio of the pore volume, $V_{p}$, to the total sample volume, $V_{t}$. Porosity is defined as [13,14]:

(3) $$n=\frac{V_{p}}{V_{t}}$$

The degree of saturation can be determined by rearranging Equation (2) into the following form:

(4) $$S_{r}=\frac{V_{w}}{n\cdot V_{t}}.$$

Assuming that water is present only in the pore space, the total sample volume, $V_{t}$, is equal to the sand sample volume, $V_{s}$. Hence:

(5) $$S_{r}=\frac{V_{w}}{n\cdot V_{s}}.$$

Considering that the densities of water, $\rho_{w}$, and sand, $\rho_{s}$, are defined in terms of the water mass, $m_{w}$, the sand mass, $m_{s}$, and their corresponding volumes are calculated as follows:

(6) $$\rho_{w}=\frac{m_{w}}{V_{w}},$$

(7) $$\rho_{s}=\frac{m_{s}}{V_{s}}.$$

Equation (5) can be rewritten as:

(8) $$S_{r}=\frac{\rho_{s}}{\rho_{w}\cdot n}\cdot \frac{m_{w}}{m_{s}}.$$

### 3.2. Effect of Moisture Content on the Spectral Characteristics of Sand

**Figure 3:**
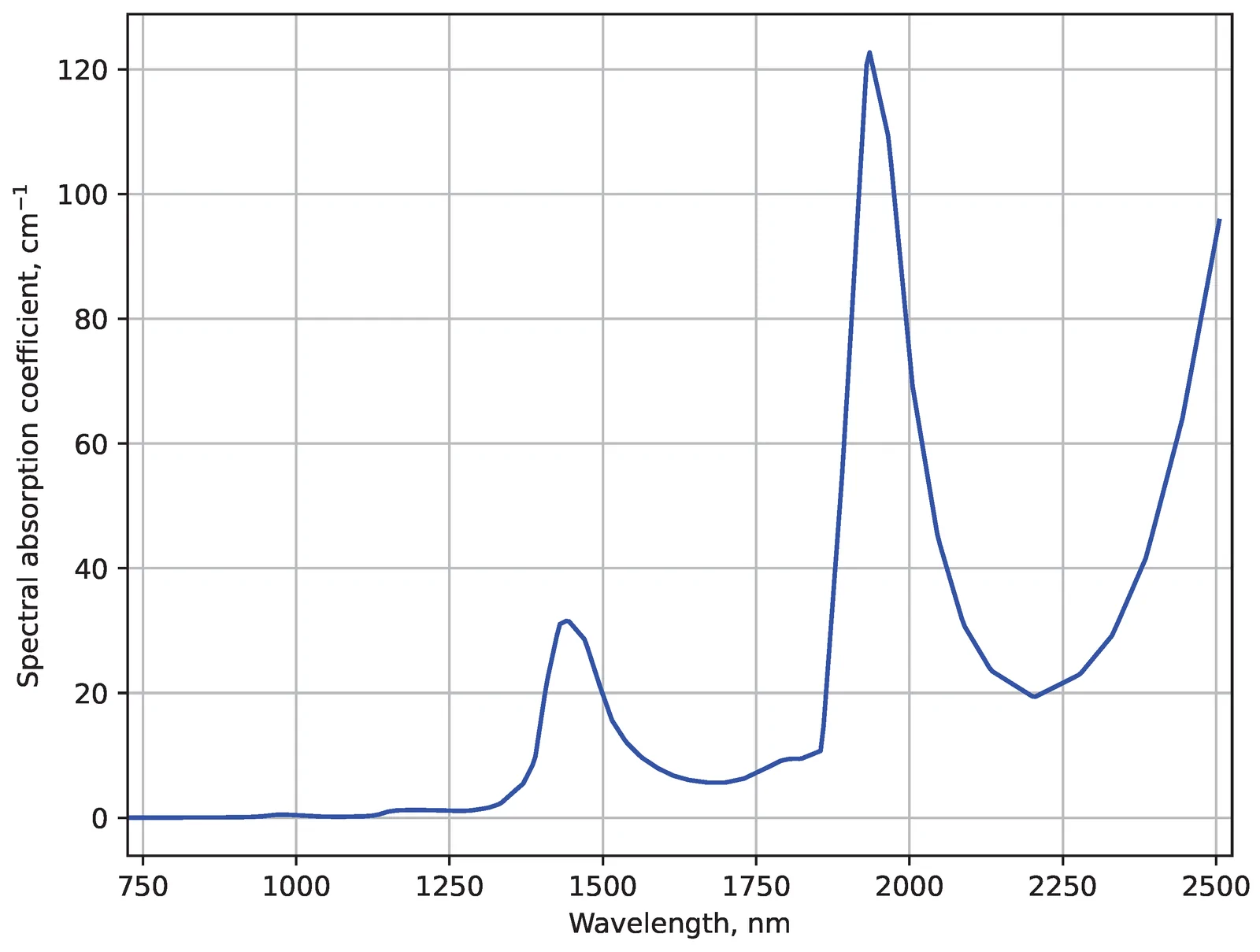
Infrared absorption spectrum of water.

Changes in the moisture content of sand affect its spectral response in the infrared region. This effect results from the increased absorption of electromagnetic radiation by water molecules. Within the analyzed spectral range, the recorded radiance decreases with increasing water content. This phenomenon is most pronounced in spectral regions corresponding to the characteristic absorption bands of water. These changes enable the quantitative estimation of the water content. Figure 3 presents the infrared absorption spectrum of water based on the reported data [7].

In the proposed method, the analysis was limited to the near-infrared (NIR) spectral range. Particular attention was given to the 1400–1500 nm region, which encompasses a strong water absorption band associated with O–H bond vibrations [15].

### 3.3. Radiometric Model of Moisture Measurement

To develop a model for determining the moisture content of sand using hyperspectral imaging in the infrared spectral range, it is necessary to identify all components of the radiation reaching the imaging spectrometer. In addition to the infrared radiation emitted or reflected by the observed object, the imaging spectrometer also receives radiation originating from surrounding objects (e.g., people, trees, buildings, and other environmental objects), as well as radiation reflected from the object surface [16,17,18,19]. The spectrometer also receives solar radiation, particularly that reflected from the object and surrounding objects. All radiation components are attenuated by the atmosphere along the propagation path.

To obtain accurate spectral radiance measurements using an imaging spectrometer, it is necessary to account for the influence of various physical phenomena and radiation sources that interfere with the measurement. Since many of these disturbances are difficult to estimate, mathematical models used for the analysis of the recorded infrared radiation are usually simplified in order to achieve the required accuracy while minimizing the number of parameters included in the model. Partial compensation for these disturbances can be achieved using the following information [3,16,17,18,19]:

- Object emissivity;
- Ambient temperature;
- Distance to the observed object;
- Relative atmospheric humidity.

In addition, the influence of the following factors on the measured radiance of the observed object is often compensated for [18,19,20]:

- The temperature of the optical components of the objective (including, for example, optical windows);
- The transmittance of the optical components;
- Atmospheric temperature and transmittance;
- The internal temperature of the spectrometer.

**Figure 4:**
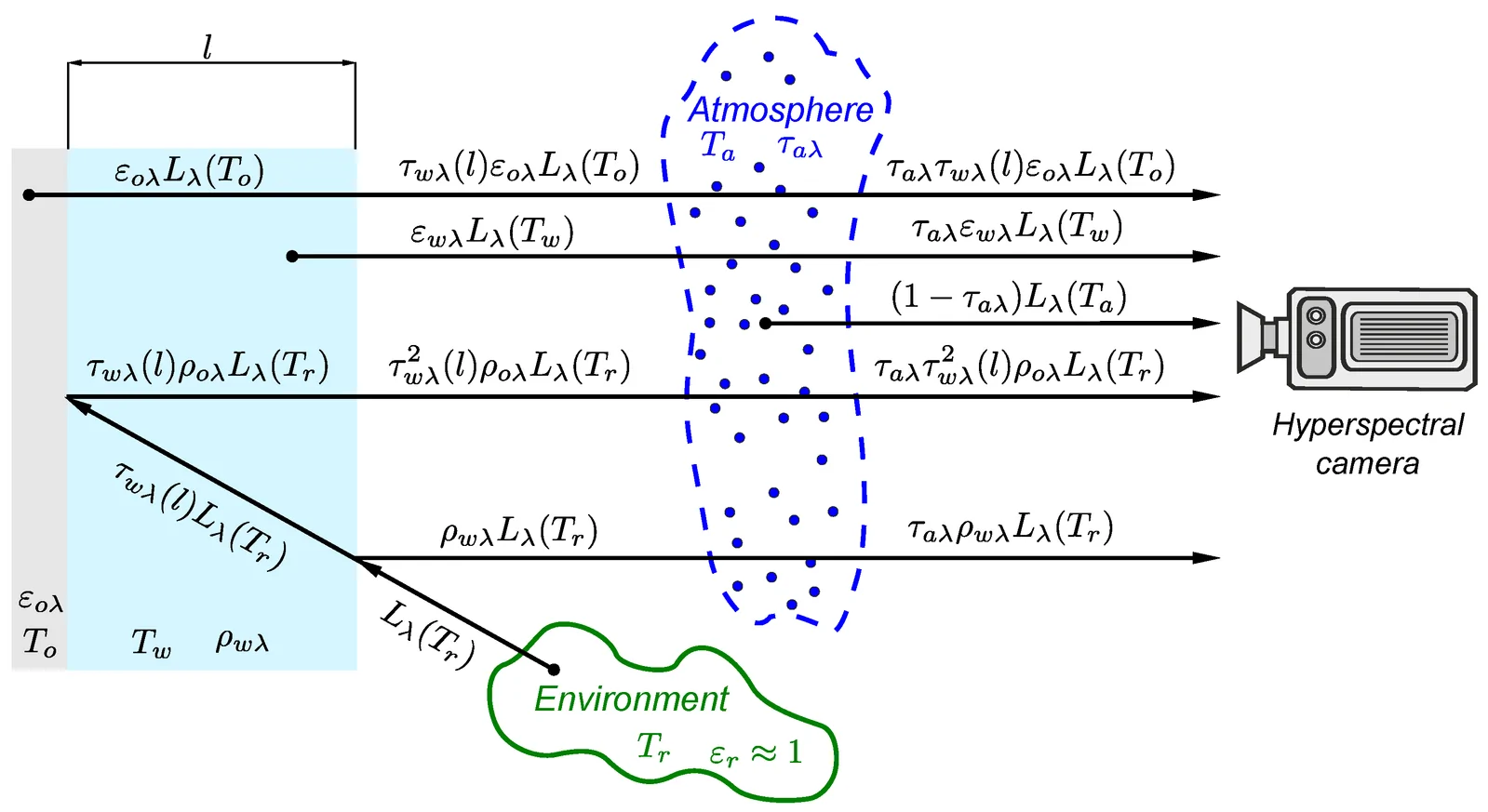
Radiometric model of a scene for hyperspectral soil moisture measurements.

It should be noted that the analysis of spectrometric data requires consideration of numerous physical phenomena, including power supply noise, detector noise, detector temperature, ambient temperature, the speed and method of digital data processing, and the parameters of analog-to-digital conversion [18,20]. Accounting for all these phenomena at the highest level of detail is generally impractical or prohibitively expensive. Therefore, a compromise between measurement accuracy and the cost of system development and implementation is usually adopted, which is reflected in the achievable system performance. In thermal imaging systems, it is commonly assumed that the infrared radiation reaching the spectrometer originates from the sources shown in Figure 4 [16,18,19,20].

According to the notation shown in Figure 4, the total spectral radiance reaching the spectrometer objective consists of several fundamental radiation components and can be expressed as:

(9) $$L_{C\lambda }=W_{o\lambda }+W_{w\lambda }+W_{ro\lambda }+W_{rw\lambda }+W_{a\lambda },$$

where: $W_{o\lambda }$ is the spectral radiant power density of the object (sand), $W_{w\lambda }$ is the spectral radiant power density of water, $W_{ro\lambda }$ is the spectral radiant power density of the radiation reflected from the object (sand), $W_{rw\lambda }$ is the spectral radiant power density of the radiation reflected from the water layer, and $W_{a\lambda }$ is the spectral radiant power density of atmospheric radiation.

The spectral radiant power density emitted by the object (sand), $W_{o\lambda }$, can be expressed as:

(10) $$W_{o\lambda }=\tau_{a\lambda }\tau_{w\lambda }\left(l\right)\varepsilon_{o\lambda }L_{\lambda }\left(T_{o}\right),$$

where: $\varepsilon_{o\lambda }$ is the spectral emissivity of the object, $\tau_{w\lambda }\left(l\right)$ is the spectral transmittance of a water layer of thickness *l*, $\tau_{a\lambda }$ is the atmospheric transmittance, $T_{o}$ is the object temperature, and $L_{\lambda }\left(T_{o}\right)$ is the spectral radiance of a blackbody at temperature $T_{o}$.

The spectral radiant power density emitted by the water, $W_{w\lambda }$, can be expressed as:

(11) $$W_{w\lambda }=\tau_{a\lambda }\varepsilon_{w\lambda }L_{\lambda }\left(T_{w}\right),$$

where: $\varepsilon_{w\lambda }$ is the spectral emissivity of water, and $T_{w}$ is the water temperature.

The spectral radiant power density of the radiation reflected from the object, $W_{ro\lambda }$, originating from surrounding radiation sources (illumination), can be expressed as:

(12) $$W_{ro\lambda }=\tau_{a\lambda }\tau_{w\lambda }^{2}\left(l\right)\rho_{o\lambda }L_{\lambda }\left(T_{r}\right),$$

where: $\rho_{o\lambda }$ is the spectral reflectance of the object, which for opaque objects is given by $\rho_{o\lambda }=1-\varepsilon_{o\lambda }$. $T_{r}$ is the temperature of the surrounding radiation sources, where the surroundings are assumed to behave as a blackbody at temperature $T_{r}$.

The spectral radiant power density of the radiation reflected from the water surface, $W_{rw\lambda }$, originating from surrounding radiation sources, can be expressed as:

(13) $$W_{rw\lambda }=\tau_{a\lambda }\rho_{w\lambda }L_{\lambda }\left(T_{r}\right),$$

where: $\rho_{w\lambda }$ is the spectral reflectance of water.

The spectral radiant power density of atmospheric radiation, $W_{a\lambda }$, can be expressed as:

(14) $$W_{a\lambda }=\left(1-\tau_{a\lambda }\right)L_{\lambda }\left(T_{a}\right),$$

where: $\left(1-\tau_{a\lambda }\right)$ is the atmospheric emissivity (assuming that $\rho_{a\lambda }=0$), and $T_{a}$ is the atmospheric temperature.

The total spectral radiant power density reaching the hyperspectral camera from the observed scene can be expressed as:

(15) $$\begin{array}{ccc} L_{C\lambda } & = & \tau_{a\lambda }\tau_{w\lambda }\left(l\right)\varepsilon_{o\lambda }L_{\lambda }\left(T_{o}\right)+\tau_{a\lambda }\varepsilon_{w\lambda }L_{\lambda }\left(T_{w}\right)+\tau_{a\lambda }\tau_{w\lambda }^{2}\left(l\right)\rho_{o\lambda }L_{\lambda }\left(T_{r}\right)+\\ & & \tau_{a\lambda }\rho_{w\lambda }L_{\lambda }\left(T_{r}\right)+\left(1-\tau_{a\lambda }\right)L_{\lambda }\left(T_{a}\right). \end{array}$$

Assuming that, for the wavelength range of 0.9–1.7 µm and for

$$T_{o}=T_{w}=T_{a}\approx 300\;K,$$

$$T_{r}\approx 3000\;K$$

the following assumptions are valid:

(16) $$L_{\lambda }\left(T_{r}\right)\gg L_{\lambda }\left(T_{o}\right),\;L_{\lambda }\left(T_{r}\right)\gg L_{\lambda }\left(T_{w}\right),\;L_{\lambda }\left(T_{r}\right)\gg L_{\lambda }\left(T_{a}\right),$$

Equation (15) can be rewritten as follows:

(17) $$L_{C\lambda }=\left[\tau_{w\lambda }^{2}\left(l\right)\rho_{o\lambda }+\rho_{w\lambda }\right]\tau_{a\lambda }L_{\lambda }\left(T_{r}\right).$$

Since:

(18) $$\rho_{w\lambda }=1-\tau_{w\lambda }\left(l\right)-\alpha_{w\lambda },$$

then Equation (17) can be rewritten as:

(19) $$L_{C\lambda }=\left[\tau_{w\lambda }^{2}\left(l\right)\rho_{o\lambda }-\tau_{w\lambda }\left(l\right)+1-\alpha_{w\lambda }\right]\tau_{a\lambda }L_{\lambda }\left(T_{r}\right).$$

Prior to further spectral analysis, the recorded spectra were smoothed using the Savitzky–Golay (SG) filter. This method is widely used for preprocessing hyperspectral data because it reduces high-frequency noise while preserving the characteristic shape of the spectral curve and its absorption features. The SG filter performs a local polynomial approximation of the spectral signal using the least-squares method within a moving window. The smoothed value at the central point of the window is obtained from a weighted combination of the neighboring spectral values [21]. The smoothed value of the spectral signal is given by:

(20) $$L_{Fj}=\frac{\sum_{i=-m}^{m}C_{i}L_{Cj+i}}{N},$$

where $L_{F,j}$ is the filtered spectral radiance at the *j*-th spectral channel, $L_{C,j+i}$ denotes the original spectral radiance at the neighboring spectral channels within the moving window, $C_{i}$ denotes the convolution coefficient determined by the polynomial least-squares fitting, *m* is the half-width of the smoothing window, and N=2m+1 is the number of spectral data points included in the window.

To determine the radiant power recorded within the wavelength range $\lambda \;\in \;\langle \lambda_{1},\lambda_{2}\rangle$, Equation (19) is integrated over the corresponding spectral interval, yielding:

(21) $$L_{F}=\int_{\lambda_{1}}^{\lambda_{2}}\left[\tau_{w\lambda }^{2}\left(l\right)\rho_{o\lambda }-\tau_{w\lambda }\left(l\right)+1-\alpha_{w\lambda }\right]\tau_{a\lambda }L_{\lambda }\left(T_{r}\right)d\lambda .$$

Both the value of $L_{C\lambda }$ (calculated using Equation (19)) and the value of $L_{F}$ (calculated using Equation (21)) depend on the transmittance of radiation through water, $\tau_{w\lambda }\left(l\right)$. Since the transmittance of water depends on the thickness of the water layer, *l*, the recorded radiant power from the sand sample is related to its moisture content. However, both $L_{C\lambda }$ and $L_{F}$ also depend on the power of the incident radiation illuminating the object, represented by $L_{\lambda }\left(T_{r}\right)$. Under real measurement conditions (outside the laboratory), the incident radiation varies uncontrollably over a wide range of values. Consequently, the direct application of the above equations under field conditions may result in significant measurement errors. To potentially reduce the influence of changes in illumination intensity, a moisture coefficient was defined as the ratio of the recorded radiant power in two selected spectral ranges. The moisture coefficient for the spectral ranges $\Delta \lambda_{A}$ (for $\lambda \in \langle \lambda_{A1},\lambda_{A2}\rangle$) and $\Delta \lambda_{B}$ (for $\lambda \in \langle \lambda_{B1},\lambda_{B2}\rangle$) is defined as:

(22) $$W_{r}=\frac{\int_{\lambda_{A1}}^{\lambda_{A2}}\left[\tau_{w\lambda }^{2}\left(l\right)\rho_{o\lambda }-\tau_{w\lambda }\left(l\right)+1-\alpha_{w\lambda }\right]\tau_{a\lambda }L_{\lambda }\left(T_{r}\right)d\lambda }{\int_{\lambda_{B1}}^{\lambda_{B2}}\left[\tau_{w\lambda }^{2}\left(l\right)\rho_{o\lambda }-\tau_{w\lambda }\left(l\right)+1-\alpha_{w\lambda }\right]\tau_{a\lambda }L_{\lambda }\left(T_{r}\right)d\lambda }.$$

The proper selection of the spectral ranges $\Delta \lambda_{A}$ and $\Delta \lambda_{B}$ is essential for obtaining a reliable moisture coefficient. The spectral range $\Delta \lambda_{A}$ should be selected so that the attenuation of radiation by water is as low as possible. In contrast, the spectral range $\Delta \lambda_{B}$ should be selected so that the attenuation of radiation by water is as high as possible. For such a selection of spectral ranges, the transmittance of water within the spectral range $\Delta \lambda_{A}$ remains relatively high and, according to the Lambert–Beer law, depends on the thickness of the water layer, *l*. In contrast, for the spectral range $\Delta \lambda_{B}$, the water transmittance satisfies $\tau_{w\lambda }\left(l\right)\approx 0$. Consequently, Equation (22) can be rewritten as:

(23) $$W_{r}=\frac{\int_{\lambda_{A1}}^{\lambda_{A2}}\left[\tau_{w\lambda }^{2}\left(l\right)\rho_{o\lambda }-\tau_{w\lambda }\left(l\right)+1-\alpha_{w\lambda }\right]\tau_{a\lambda }L_{\lambda }\left(T_{r}\right)d\lambda }{\int_{\lambda_{B1}}^{\lambda_{B2}}\left[1-\alpha_{w\lambda }\right]\tau_{a\lambda }L_{\lambda }\left(T_{r}\right)d\lambda }.$$

Furthermore, it can be observed that for the wavelength range of 0.9–1.7 µm and for $T_{r}\approx 3000\;K$, the following assumptions are valid:

$$\tau_{a\lambda }\approx \mathrm{const},\;\alpha_{w}=\alpha_{w\lambda }\approx \mathrm{const},\;L_{\lambda }\left(T_{r}\right)\approx \mathrm{const}.$$

That is, these quantities are assumed to be independent of wavelength. Under these assumptions, Equation (23) can be rewritten as:

(24) $$W_{r}\approx \frac{1}{\left(1-\alpha_{w}\right)\left(\lambda_{B2}-\lambda_{B1}\right)}\int_{\lambda_{A1}}^{\lambda_{A2}}\left[\tau_{w\lambda }^{2}\left(l\right)\rho_{o\lambda }-\tau_{w\lambda }\left(l\right)+1-\alpha_{w}\right]d\lambda .$$

It follows from the above considerations that the moisture coefficient, $W_{r}$, depends primarily on the water transmittance, $\tau_{w\lambda }\left(l\right)$. The transmittance of an absorbing medium is described by the Lambert–Beer law [22,23]:

(25) $$\tau_{w\lambda }\left(l\right)=e^{-k_{\lambda }l}$$

where: $k_{\lambda }$ is the water absorption coefficient, m^−1^, and *l* is the thickness of the absorbing medium (water), m.

It follows from Equations (24) and (25) that the moisture coefficient, $W_{r}$, exhibits an exponential dependence on the water layer thickness and, consequently, on the moisture content of the material. The exponential nature of this relationship is also confirmed by experimental studies [24,25,26].

Therefore, it was assumed that the influence of moisture on the recorded hyperspectral signal can be approximated by the following function:

(26) $$W_{r}=ae^{bS_{r}}+c,$$

where $W_{r}$ is the moisture coefficient determined from the recorded spectral signal according to Equation (23), while $S_{r}$ is the moisture content expressed as the degree of saturation. Parameter *a* determines the amplitude of the exponential components, *b* is the attenuation coefficient, and *c* is the offset parameter.

For known values of the parameters *a*, *b*, and *c*, the moisture content of the observed sand sample can be determined directly by applying the inverse function of Equation (26). Because the exponential model is monotonic over the considered moisture range, a unique inverse function exists. Consequently, the degree of saturation is calculated as

(27) $$S_{r}=\frac{1}{b}\ln\left(\frac{W_{r}-c}{a}\right).$$

## 4. Experimental Setup

### 4.1. Procedure for Preparing Samples with a Specified Moisture Content

**Figure 5:**
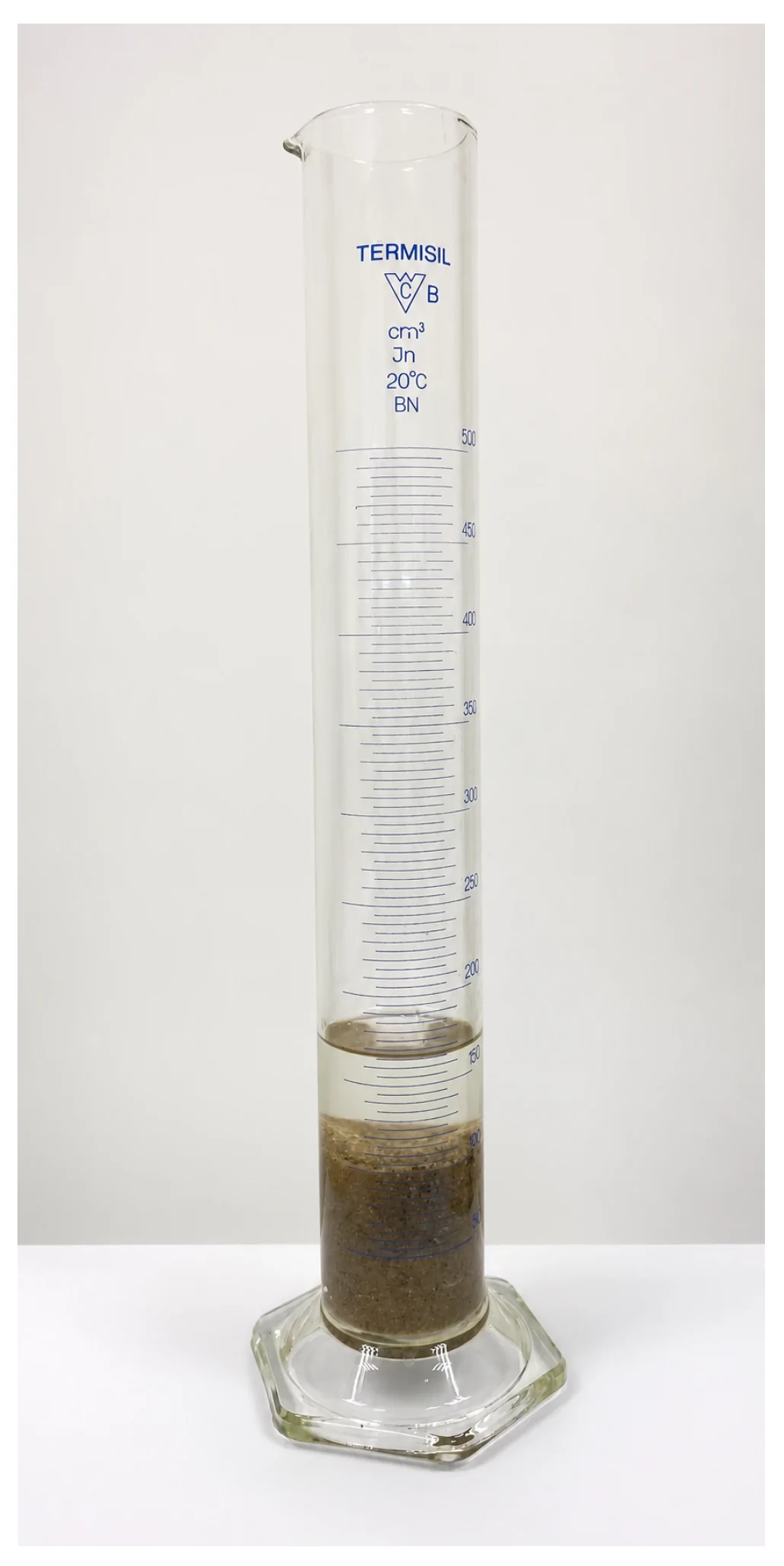
Sand sample in a graduated cylinder during pore volume determination.

To calibrate the hyperspectral moisture measurement system (i.e., to determine the parameters *a*, *b*, and *c*) and to verify its performance and accuracy, reference sand samples with known moisture contents were required. Therefore, a procedure was developed for determining the model parameters and preparing such reference samples. In the experiment, sand with a particle size ranging from 0 to 1.8 mm was used. To remove residual moisture, the sand was dried in an oven at 160 °C for 3 h. Initially, the volume of the void spaces between the sand grains, i.e., the pore volume, was determined. For this purpose, 100 cm^3^ of dry sand was measured using a graduated cylinder, after which 100 cm^3^ of water was added. Following the addition of water, air bubbles were observed escaping from the pore spaces as the trapped air was gradually displaced. After the system reached equilibrium, the final volume of the sand–water mixture was measured to be 165 cm^3^. This procedure is illustrated in Figure 5.

Assuming that the water completely fills the void spaces between the sand grains, the volume of the sand grains remains constant, and no water is absorbed into the grains, the pore volume of the sand can be determined as follows:

(28) $$V_{p}=\left(V_{s}+V_{w}\right)-V_{k}=\left(100+100\right)-165=35\;{\mathrm{cm}}^{3}$$

where: $V_{p}$ is the pore volume (void space) of the sand sample, $V_{s}$ is the initial volume of dry sand measured in the graduated cylinder, $V_{w}$ is the volume of water added to the cylinder to fill the pore spaces, and $V_{k}$ is the final measured volume of the sand–water mixture after the air bubbles had been removed.

After determining the pore volume, the porosity was calculated according to Equation (3):

(29) $$n=\frac{35}{100}=0.35.$$

The prepared sample was then weighed using a laboratory balance, yielding a sample mass of $m_{s}=170\;g$. The bulk density of the sand was subsequently calculated according to Equation (7), resulting in a value of $\rho_{s}=1.7\;g\cdot {\mathrm{cm}}^{-3}$.

The determined parameters were subsequently used to calculate the amount of water required to obtain the desired moisture content. The required water mass was calculated using Equation (8), which can be rearranged as:

(30) $$m_{w}=\frac{S_{r}\cdot \rho_{w}\cdot n\cdot m_{s}}{\rho_{s}}.$$

The procedure for preparing a sand sample with the desired moisture content is as follows:

1. Place dry sand (previously dried in an oven) into a container ($m_{s}=510\;g$).
2. Calculate the required water mass $m_{w}$ needed to achieve the desired moisture content $S_{r}$ using Equation (30) and the previously determined parameters.
3. Add the previously calculated amount of water to the sand sample.
4. Thoroughly mix the sand and water until a homogeneous mixture is obtained.
5. Perform a control weighing of the prepared sample.

Immediately before the hyperspectral measurements, each prepared sand–water sample was weighed again to verify that the target moisture content had been maintained throughout the experiment. Monitoring the sample mass enabled continuous assessment of water losses caused by evaporation and ensured that the desired moisture content was maintained by replenishing any water deficit as required.

### 4.2. Experimental Setup for Parameter Estimation, Calibration, and System Validation

During sand moisture measurements based on Equation (27), the moisture coefficient $W_{r}$ is determined using the approximation function given in Equation (26). The application of the approximation function requires prior determination of its parameters, namely *a*, *b*, and *c*. To determine the values of these parameters, a dedicated experimental setup was developed together with proprietary software for data acquisition, analysis, and processing. The experimental setup consists of the following main components:

- A hyperspectral camera;
- An illumination system;
- A set of sand samples with different moisture contents (placed on the scanning table);
- A motorized scanning table with its controller;
- A computer equipped with software for system control, data acquisition, analysis, and processing.

**Figure 6:**
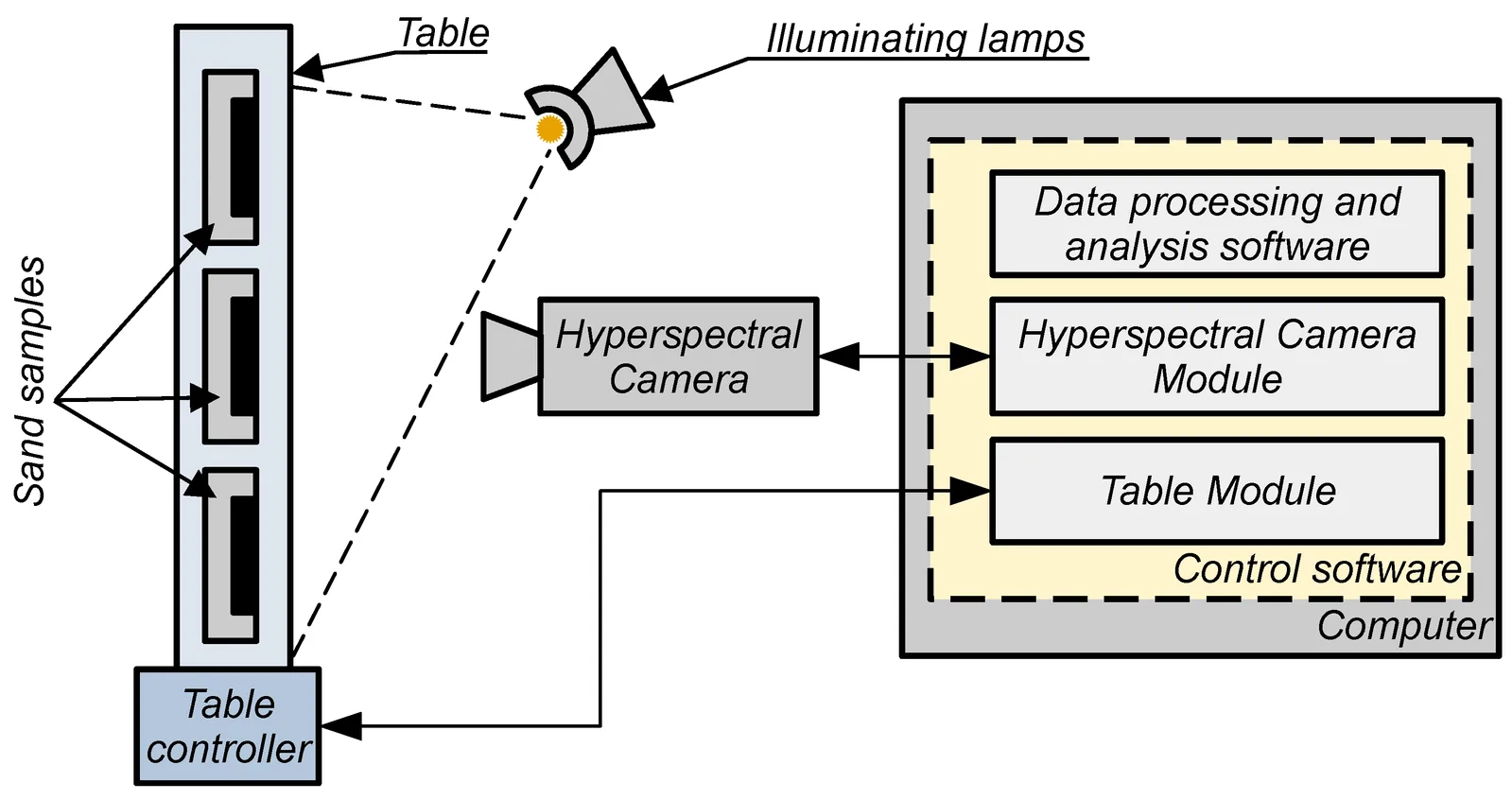
Schematic diagram of the experimental setup for moisture determination.

**Figure 7:**
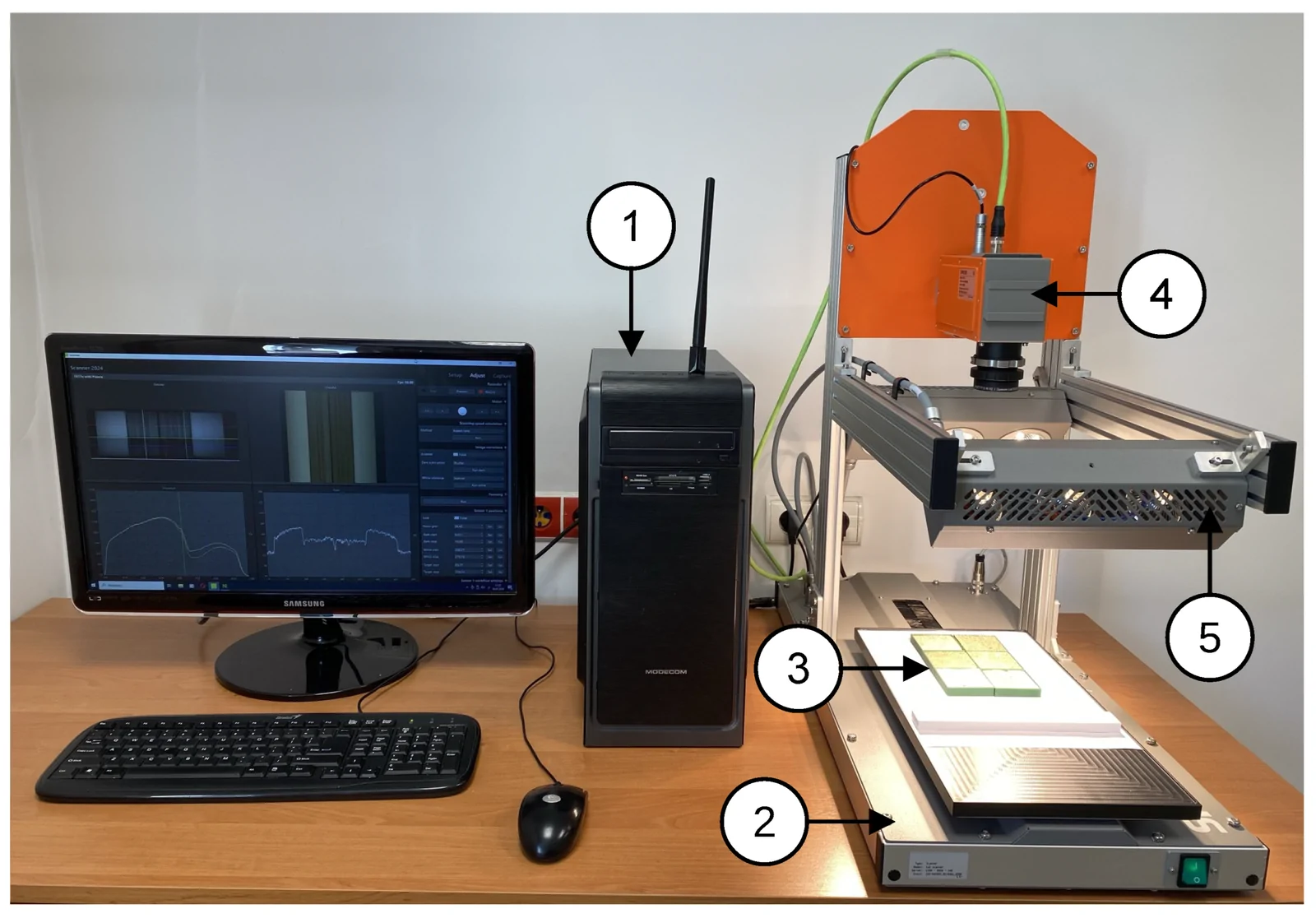
View of the experimental setup used for the calibration and validation of the moisture measurement system, consisting of a computer (1), a motorized scanning table with its controller (2), sand samples (3), a hyperspectral camera (4), and an illumination system (5).

The block diagram of the experimental setup used for data acquisition and parameter estimation is shown in Figure 6, while a photograph of the experimental setup during hyperspectral measurements of the sand samples is presented in Figure 7.

The most important component of the experimental setup is the hyperspectral camera, which is used to acquire the spectral distribution of the investigated samples. A Specim FX17 hyperspectral camera manufactured by SPECIM was used in the experimental setup [27]. The camera operates in the near-infrared (NIR) spectral range and enables the acquisition of hyperspectral images in multiple narrow spectral bands. The technical specifications of the Specim FX17 camera (Specim, Spectral Imaging Ltd., Oulu, Finland) are presented in Table 1.

The Specim FX17 is a pushbroom hyperspectral camera. Therefore, the experimental setup incorporates a motorized scanning table with a dedicated controller to provide controlled movement of the sample relative to the camera. Translating the sample during image acquisition enables the reconstruction of a hyperspectral image (hyperspectral data cube). The laboratory setup was equipped with a LabScanner system, consisting of a motorized scanning table, a controller, and dedicated software [28]. An artificial illumination system was mounted on the scanner frame to provide stable and uniform lighting conditions during the measurements. The technical specifications of the LabScanner system are presented in Table 2.

**Figure 8:**
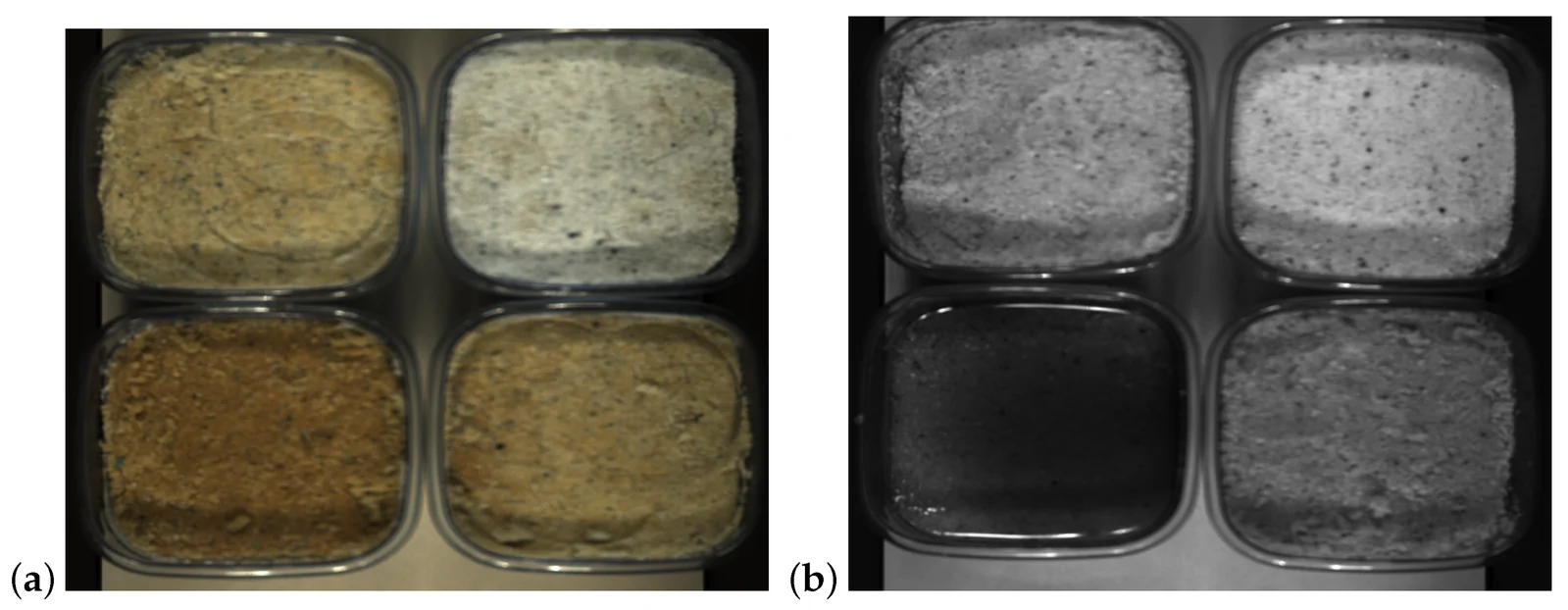
Photographs of sand samples (**a**) with different moisture contents and the corresponding hyperspectral image (**b**) acquired at a wavelength of 1200 nm.

A key component of the experimental setup for parameter estimation, calibration, and system validation is the control software. Hyperspectral data acquired by the Specim FX17 camera are transmitted to and recorded by the control software via an Ethernet interface. The use of the Ethernet interface enables direct data transfer to the computer without the need for additional hardware. The computer is also used to control the motorized scanning table, allowing precise movement of the sand samples during image acquisition, as well as to perform data analysis and processing using software developed in Python. An example of a moist sand sample together with its corresponding hyperspectral image is presented in Figure 8.

## 5. Uncertainty Analysis of Sand Moisture Measurements

One of the most important performance characteristics of the proposed sand moisture measurement system is its measurement uncertainty, which is reflected by the dispersion of the measured moisture values [29]. The measurement uncertainty of the developed hyperspectral moisture measurement system can be determined based on measurements performed on prepared sand samples with known moisture contents. The absolute measurement error is defined as [30]:

(31) $$\Delta S_{r}=S_{rD}-S_{rR},$$

where: $S_{rD}$ is the moisture content determined from the data acquired by the hyperspectral camera using Equation (27), $S_{rR}$ is the reference (“true”) moisture content of the calibration sand sample, and $\Delta S_{r}$ is the absolute measurement error of the moisture determination.

In the adopted method for estimating the sand moisture measurement error, the measurement uncertainty consists of the following components:

- The statistical dispersion of the measurement results ($u_{S}$), representing the Type A standard uncertainty;
- The uncertainty associated with the reference moisture content ($u_{R}$), representing the Type B standard uncertainty.

Accordingly, the uncertainty budget can be expressed in terms of the combined standard uncertainty [29] as:

(32) $$u=\sqrt{u_{S}^{2}+u_{R}^{2}}.$$

The Type A evaluation of uncertainty [29] is based on the statistical analysis of a series of *N* direct measurements (an *N*-element sample), denoted as $\Delta S_{1},\Delta S_{2},\ldots ,\Delta S_{N}$. If the measurement conditions ensure the same accuracy for all independent measurements, the result of a single measurement can be treated as a random variable following a normal (Gaussian) distribution. The measurement result is taken as the arithmetic mean of the *N* measurement values:

(33) $$\bar{S}=\frac{1}{N}\sum_{i=1}^{N}S_{i},$$

which is regarded as a reliable estimate of the true value. Under these assumptions, the Type A standard uncertainty of the measurement result can be calculated as:

(34) $$u_{S}=\sqrt{\frac{1}{N(N-1)}\sum_{i=1}^{N}{\left(S_{i}-\bar{S}\right)}^{2}},$$

The principal factor affecting the uncertainty of the moisture measurements in the adopted error analysis procedure is the reference standard used, i.e., the sand samples with known moisture contents. In this case, the Type B evaluation of uncertainty [29] is associated with the limiting uncertainty of the reference moisture content of the calibration sand samples.

The moisture content of the reference samples is determined using the gravimetric method based on Equation (2). Consequently, the uncertainty of the degree of water saturation is expressed as a combined standard uncertainty, including a covariance term, since both the water mass ($m_{w}$) and the sand mass ($m_{s}$) are measured using the same laboratory balance and their measurement uncertainties may therefore be partially correlated. The combined standard uncertainty is calculated according to [29,31,32]:

(35) $$u_{R}=\sqrt{{\left(\frac{\partial S_{r}}{\partial m_{w}}\right)}^{2}u^{2}\left(m_{d}\right)+{\left(\frac{\partial S_{r}}{\partial m_{s}}\right)}^{2}u^{2}\left(m_{s}\right)+2\frac{\partial S_{r}}{\partial m_{w}}\frac{\partial S_{r}}{\partial m_{s}}\mathrm{cov}\left(m_{w},m_{s}\right)},$$

where $u(m_{w})$ and $u(m_{s})$ denote the standard uncertainties of the water mass and sand mass measurements, respectively, and $\mathrm{cov}(m_{w},m_{s})$ is the covariance between the random variables $m_{w}$ and $m_{s}$. The partial derivatives appearing in Equation (35) are given by:

$$\frac{\partial S_{r}}{\partial m_{w}}=\frac{\rho_{s}}{\rho_{w}\cdot n}\cdot \frac{1}{m_{s}},\;\frac{\partial S_{r}}{\partial m_{s}}=-\frac{\rho_{s}}{\rho_{w}\cdot n}\cdot \frac{m_{w}}{m_{s}^{2}},\;\frac{\partial S_{r}}{\partial m_{w}}\frac{\partial S_{r}}{\partial m_{s}}=-{\left[\frac{\rho_{s}}{\rho_{w}\cdot n}\right]}^{2}\cdot \frac{m_{w}}{m_{s}^{3}}.$$

Substituting these derivatives into Equation (35) yields:

(36) $$u_{R}=\frac{\rho_{s}}{\rho_{w}\cdot n}\cdot \sqrt{\frac{u^{2}\left(m_{w}\right)}{m_{s}^{2}}+\frac{m_{w}^{2}}{m_{s}^{4}}u^{2}\left(m_{s}\right)-2\frac{m_{w}}{m_{s}^{3}}\mathrm{cov}\left(m_{w},m_{s}\right)}.$$

Instead of the covariance, the Pearson correlation coefficient $r_{ws}$ may be used:

(37) $$\mathrm{cov}\left(m_{w},m_{s}\right)=r_{ws}\;u\left(m_{w}\right)\;u\left(m_{s}\right)$$

The resulting expression is:

(38) $$u_{R}=\frac{\rho_{s}}{\rho_{w}\cdot n}\cdot \sqrt{\frac{u^{2}\left(m_{w}\right)}{m_{s}^{2}}+\frac{m_{w}^{2}}{m_{s}^{4}}u^{2}\left(m_{s}\right)-2\frac{m_{w}}{m_{s}^{3}}r_{ws}\;u\left(m_{w}\right)\;u\left(m_{s}\right)}$$

Since both the water mass and the sand mass are measured using the same laboratory balance, it is assumed that

$$u\left(m_{w}\right)=u\left(m_{s}\right)=u\left(m\right),$$

and consequently (38) can be rewritten as:

(39) $$u_{R}=u\left(m\right)\cdot \frac{\rho_{s}}{\rho_{w}\cdot n}\cdot \frac{1}{m_{s}}\sqrt{1+\frac{m_{w}^{2}}{m_{s}^{2}}-2\frac{m_{w}}{m_{s}}r_{ws}}$$

If the calibration uncertainty of the laboratory balance is Δm, and the associated probability density function is assumed to follow a uniform (rectangular) distribution, the corresponding standard uncertainty can be calculated according to [29] as:

(40) $$u\left(m\right)=\frac{\Delta m}{\sqrt{3}}.$$

In this case, Equation (39) can be written as

(41) $$u_{R}=\frac{\Delta m}{\sqrt{3}}\cdot \frac{\rho_{s}}{\rho_{w}\cdot n}\cdot \frac{1}{m_{s}}\sqrt{1+\frac{m_{w}^{2}}{m_{s}^{2}}-2\frac{m_{w}}{m_{s}}r_{ws}}.$$

Since the experiment involves a series of paired measurements $\left(m_{w,i},m_{s,i}\right)$, the Pearson correlation coefficient $r_{ws}$ can be calculated according to [31,32] as

(42) $$r_{ws}=\frac{\sum_{i=1}^{n}\left(m_{w,i}-\bar{m_{w}}\right)\left(m_{s,i}-\bar{m_{s}}\right)}{\sqrt{\sum_{i=1}^{n}{\left(m_{w,i}-\bar{m_{w}}\right)}^{2}\;\sum_{i=1}^{n}{\left(m_{s,i}-\bar{m_{s}}\right)}^{2}}}.$$

Based on the above assumptions and Equation (32), the standard uncertainty of the sand moisture measurement obtained using the developed hyperspectral measurement system is given by

(43) $$u_{S}=\sqrt{\frac{1}{N(N-1)}\sum_{i=1}^{N}{\left(S_{i}-\bar{S}\right)}^{2}+\frac{1}{3}\cdot {\left(\frac{\rho_{s}}{\rho_{w}\cdot n}\cdot \frac{\Delta m}{m_{s}}\right)}^{2}\cdot \left(1+\frac{m_{w}^{2}}{m_{s}^{2}}-2\frac{m_{w}}{m_{s}}r_{ws}\right)},$$

The standard uncertainty *u* defines the interval from x−u to x+u, within which the true value of the measured moisture content is expected to lie. To enable comparison of moisture measurement results obtained under different experimental conditions, the expanded uncertainty *U* should be determined [29]. The expanded uncertainty is obtained by multiplying the standard uncertainty by a coverage factor so that the interval x±U contains the majority of the measured values. For the proposed hyperspectral sand moisture measurement method, the expanded uncertainty is calculated as

(44) U=k·u,

where *k* is the coverage factor. In the adopted uncertainty evaluation procedure, the probability distribution of the moisture measurement results is assumed to be approximately normal. Consequently, for a coverage factor of k=2, the corresponding confidence level is approximately 95%.

## 6. Results

As part of this study, an algorithm for determining the moisture content of sand based on the analysis and processing of hyperspectral data was developed in accordance with the proposed measurement model. The developed data processing algorithm was implemented as a Python program and deployed on the computer of the laboratory measurement system. The sand moisture content was calculated using Equation (27), which was derived from the approximation function given in Equation (26). The application of the approximation function requires prior determination of its parameters, namely *a*, *b*, and *c*.

**Figure 9:**
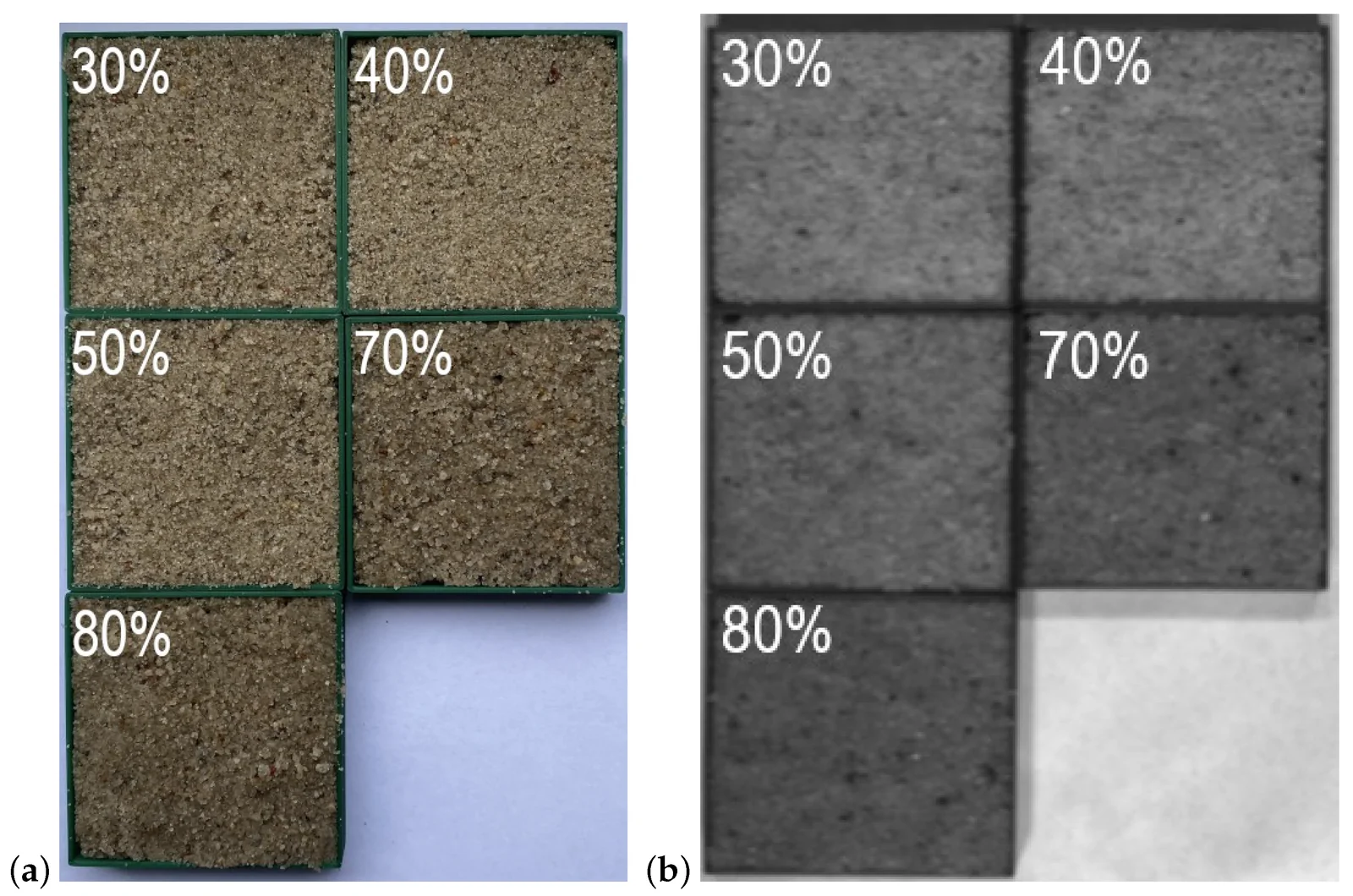
Photographs of the sand samples (**a**) with different moisture contents used for calibration and the corresponding hyperspectral image (**b**) acquired at a wavelength of 1200 nm.

To determine these parameters, the measurement system was calibrated using the laboratory setup. The calibration procedure consisted of measuring the spectral radiance $L_{C\lambda }$ for sand samples with different moisture contents $S_{r}$. The measured spectral radiance was then smoothed using the Savitzky–Golay filter according to Equation (20), yielding the filtered spectral radiance $L_{F\lambda }$. Subsequently, the moisture coefficient $W_{r}$ was calculated from the filtered spectral radiance $L_{F\lambda }$ according to Equation (22) for the spectral ranges $\Delta \lambda_{A}$ (for λ∈〈1200,1250〉) and $\Delta \lambda_{B}$ (for λ∈〈1420,1460〉). The measured objects were sand samples with different moisture contents, prepared according to the procedure described in Section 4.1. The calibration procedure yielded *K* measurement points, each consisting of a calculated value of $W_{r}$ and the corresponding moisture content $S_{r}$. The calibration was performed using five sand samples with moisture contents of 30%, 40%, 50%, 70%, and 80%. Photographs of the sand samples together with the corresponding hyperspectral image acquired at one of the analyzed spectral bands (1200 nm) are shown in Figure 9.

In this case, the primary objective is to determine the overall shape of the approximation curve that best fits the measured data points. To achieve this, the least-squares optimization method was employed. The calculations required to determine the parameters *a*, *b*, and *c* using the least-squares method were performed by means of digital data processing. The resulting values of the parameters *a*, *b*, and *c* with their corresponding 95% confidence intervals are as follows:

$$a=7.24\times 10^{-1}\in \left[6.28\times 10^{-1},\;8.20\times 10^{-1}\right].$$

$$b=-2.50\times 10^{-2}\in \left[-3.43\times 10^{-2},\;-1.57\times 10^{-2}\right].$$

$$c=1.37\times 10^{-1}\in \left[3.73\times 10^{-2},\;2.37\times 10^{-1}\right].$$

**Figure 10:**
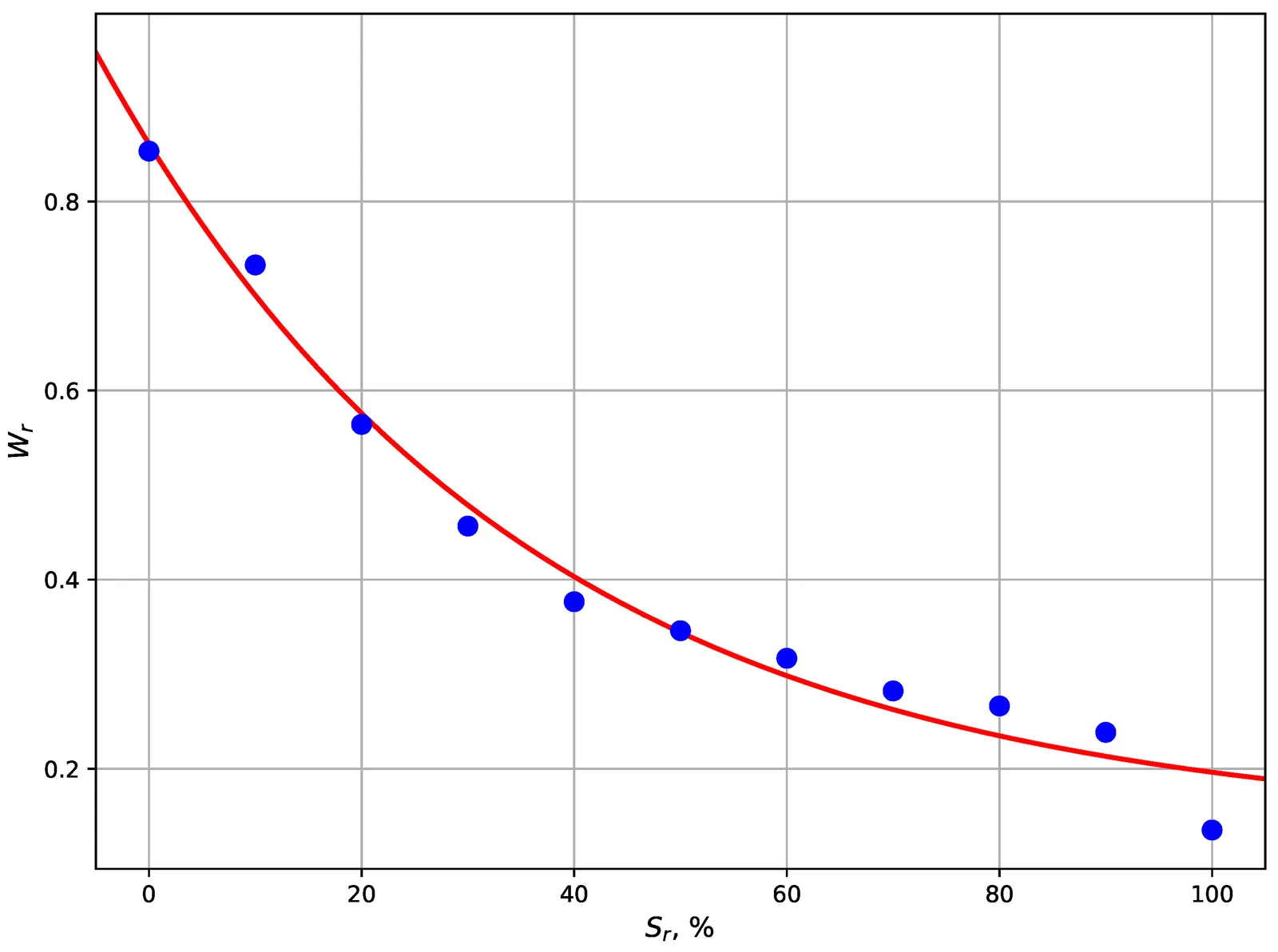
Approximation curve (red) and representative measurement points for each analyzed moisture content (blue).

To evaluate the goodness of fit of the proposed model, the coefficient of determination, $R^{2}$, was calculated. The high coefficient of determination $R^{2}=0.99$ indicates a strong fit of the exponential model to the experimental calibration data. The approximation function $W_{r}\left(S_{r}\right)$, defined by Equation (26) using the estimated parameters, is shown in Figure 10. It can be seen that the spectral response decreases nonlinearly with increasing water saturation, with a steeper decline at lower saturation levels and a more gradual decrease at higher levels. This trend indicates that the relationship between the spectral response and water saturation is not linear over the investigated range.

**Figure 11:**
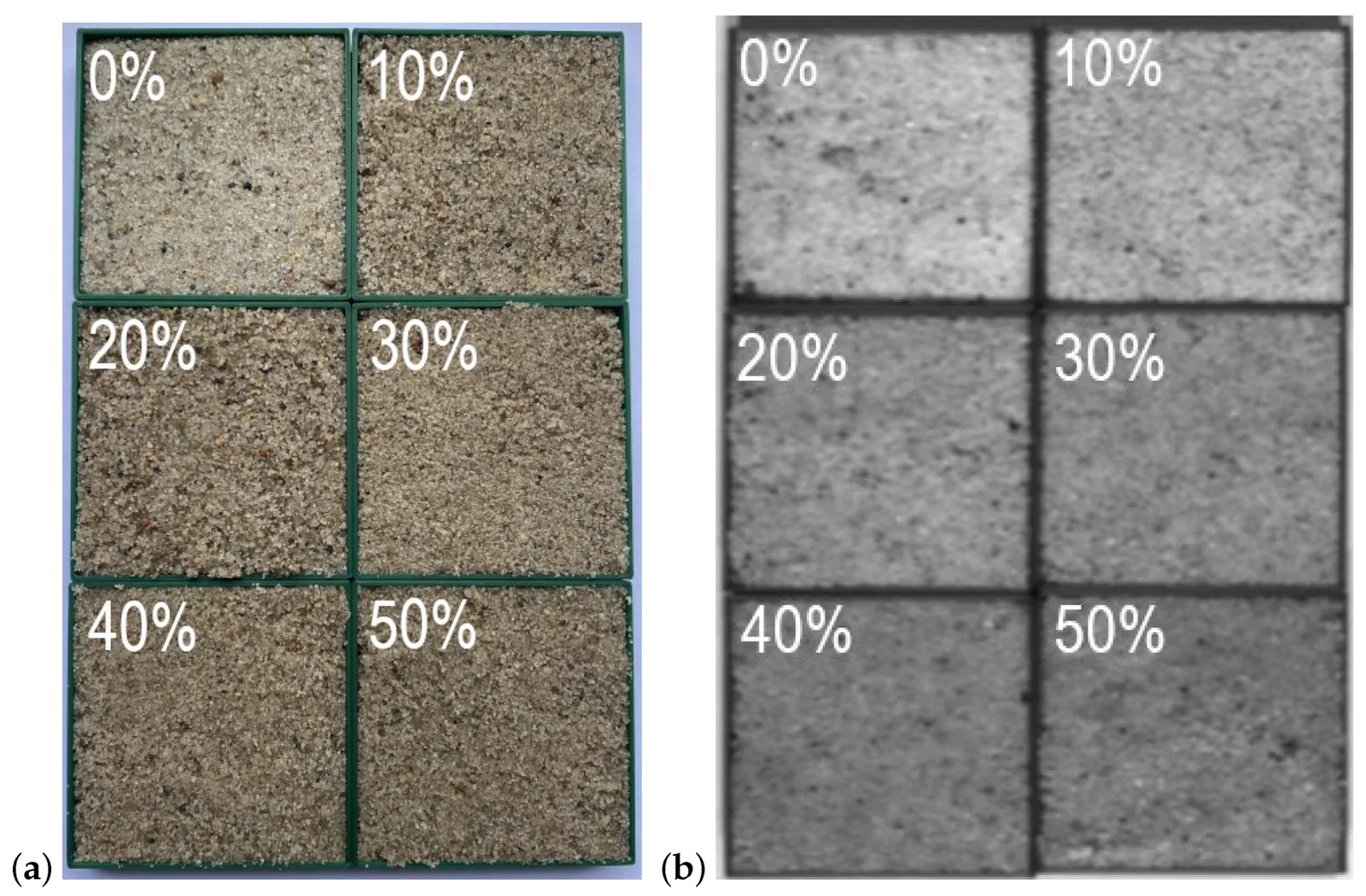
Photographs of the sand samples (**a**) with moisture contents ranging from 0% to 50% used for model validation and the corresponding hyperspectral image (**b**) acquired at a wavelength of 1200 nm.

**Figure 12:**
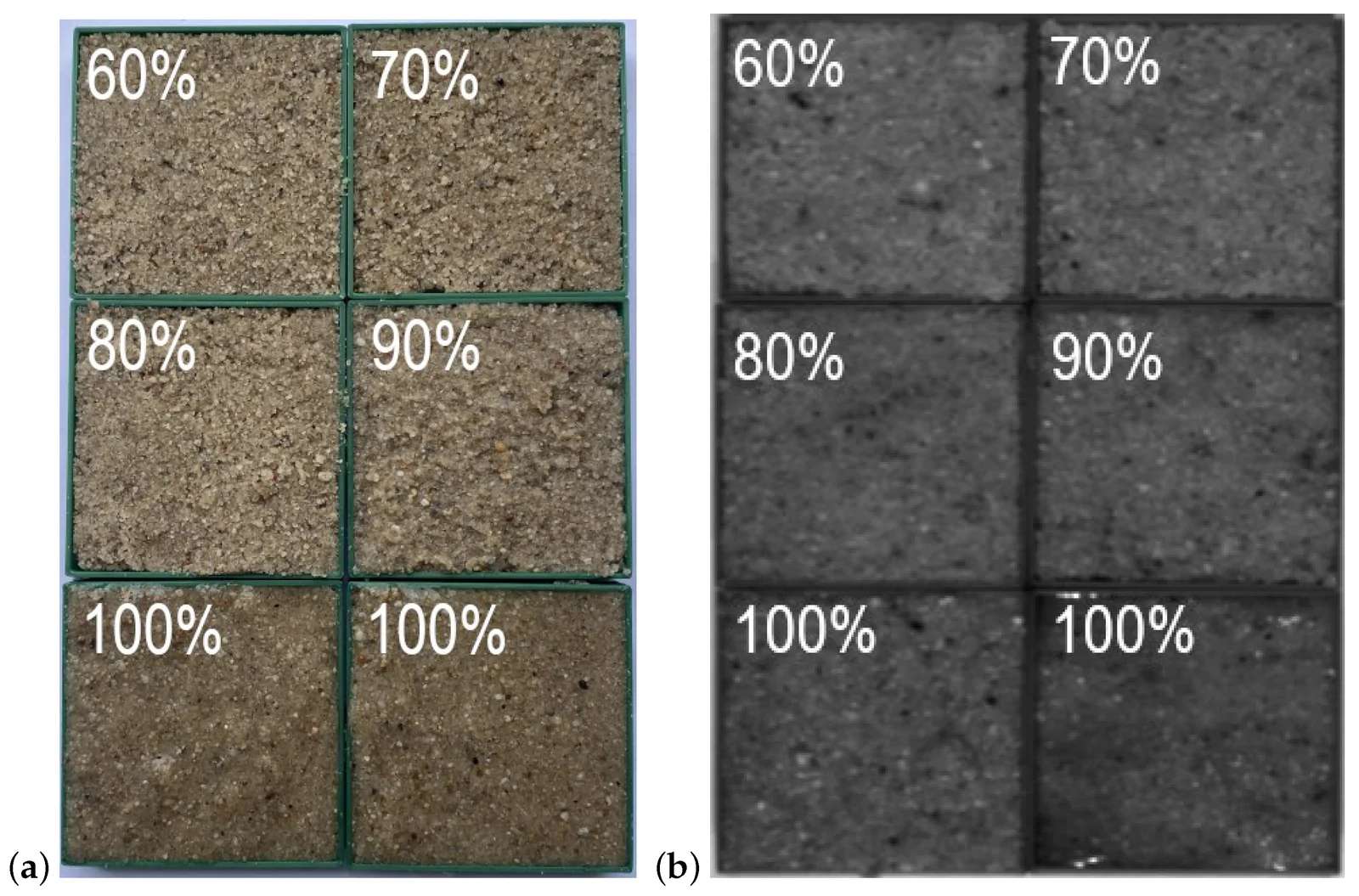
Photographs of the sand samples (**a**) with moisture contents ranging from 60% to 100% used for model validation and the corresponding hyperspectral image (**b**) acquired at a wavelength of 1200 nm.

The proposed method for determining sand moisture content from hyperspectral data acquired in the near-infrared spectral range was implemented as a Python application (version 3.12.10). The implemented algorithm was evaluated during a test measurement campaign using the laboratory experimental setup described in Section 4.2. During the experimental validation, the developed method was evaluated using hyperspectral images acquired in measurement sessions separate from those used for model calibration. The images were acquired for sand samples with different moisture contents, prepared according to the procedure described in Section 4.1. The measurement scenarios are presented in Figure 11 and Figure 12.

**Figure 13:**
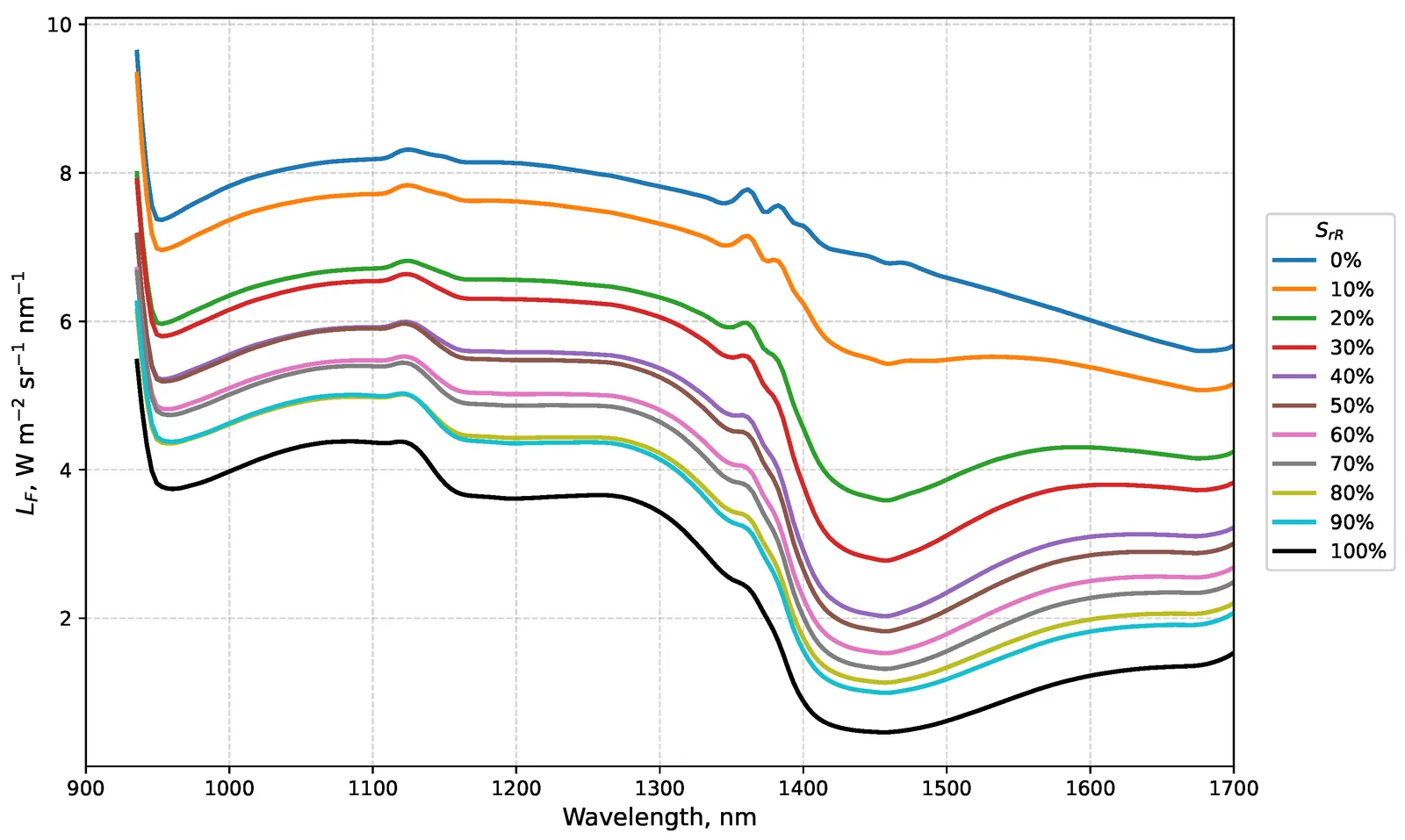
Spectral radiance as a function of wavelength for sand samples with different moisture contents.

For each sample, a region of interest (ROI), typically of size 25 × 25 pixels, was selected to account for the spatial variability in radiance associated with the heterogeneous distribution of sand and water particles. The use of an ROI enabled statistical averaging of the spectral signals, reducing the sensitivity of the method to local, random radiance variations among individual sand particles. To reduce variations in the spectral signal and obtain a smoother spectral profile while preserving its characteristic features, the Savitzky–Golay (Equation (20)) filter was applied along the wavelength dimension for each pixel within the selected ROI. A window length of 9 spectral channels and a second-order polynomial were used. After filtering, the mean spectral radiance was calculated for each wavelength within the selected ROI. This procedure yielded the spectral radiance curves for each reference sand sample. The resulting spectral curves for samples with different moisture contents are presented in Figure 13.

**Figure 14:**
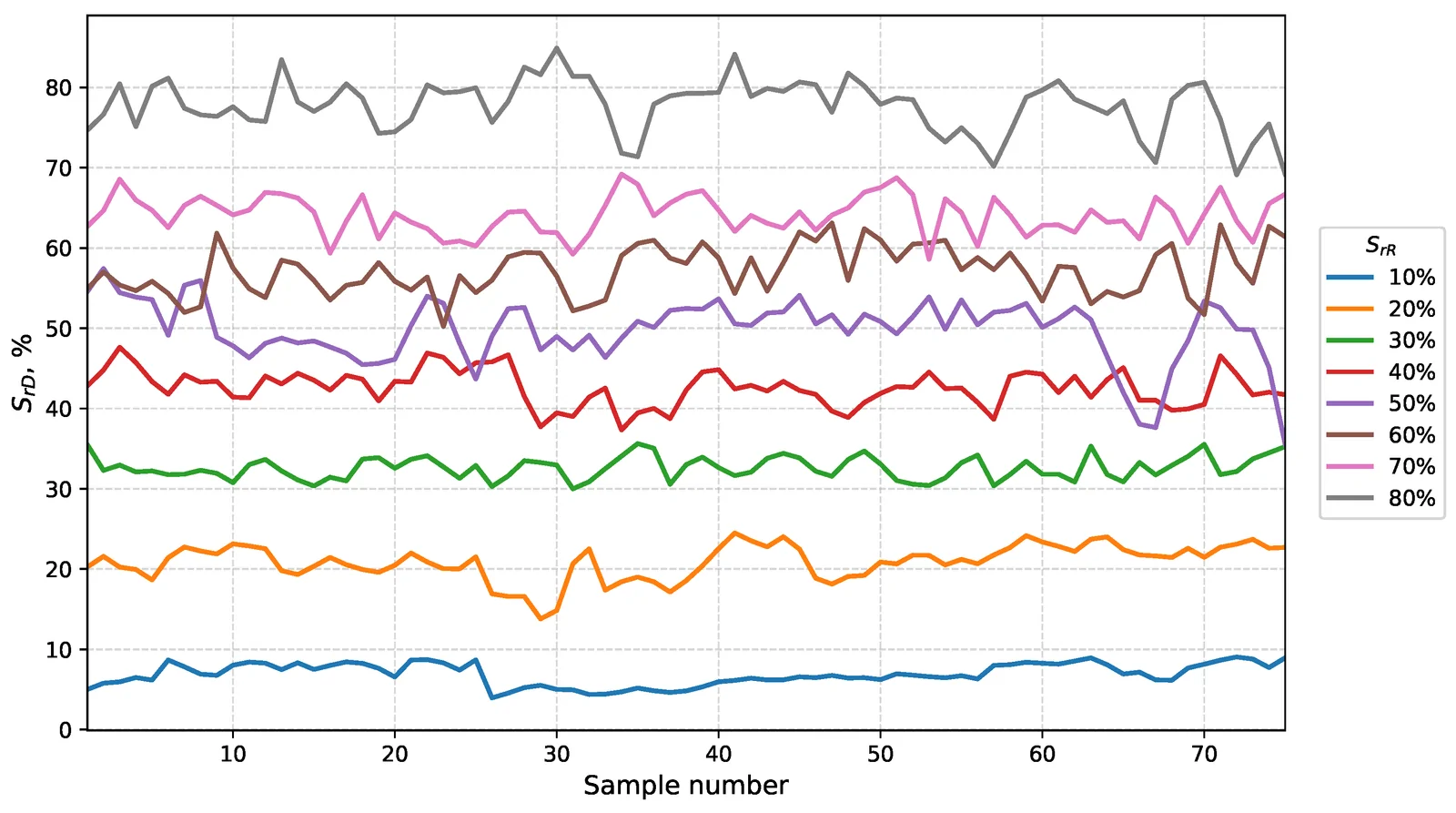
Calculated moisture content obtained from hyperspectral signal analysis for sand samples with moisture contents ranging from 10% to 80%.

**Figure 15:**
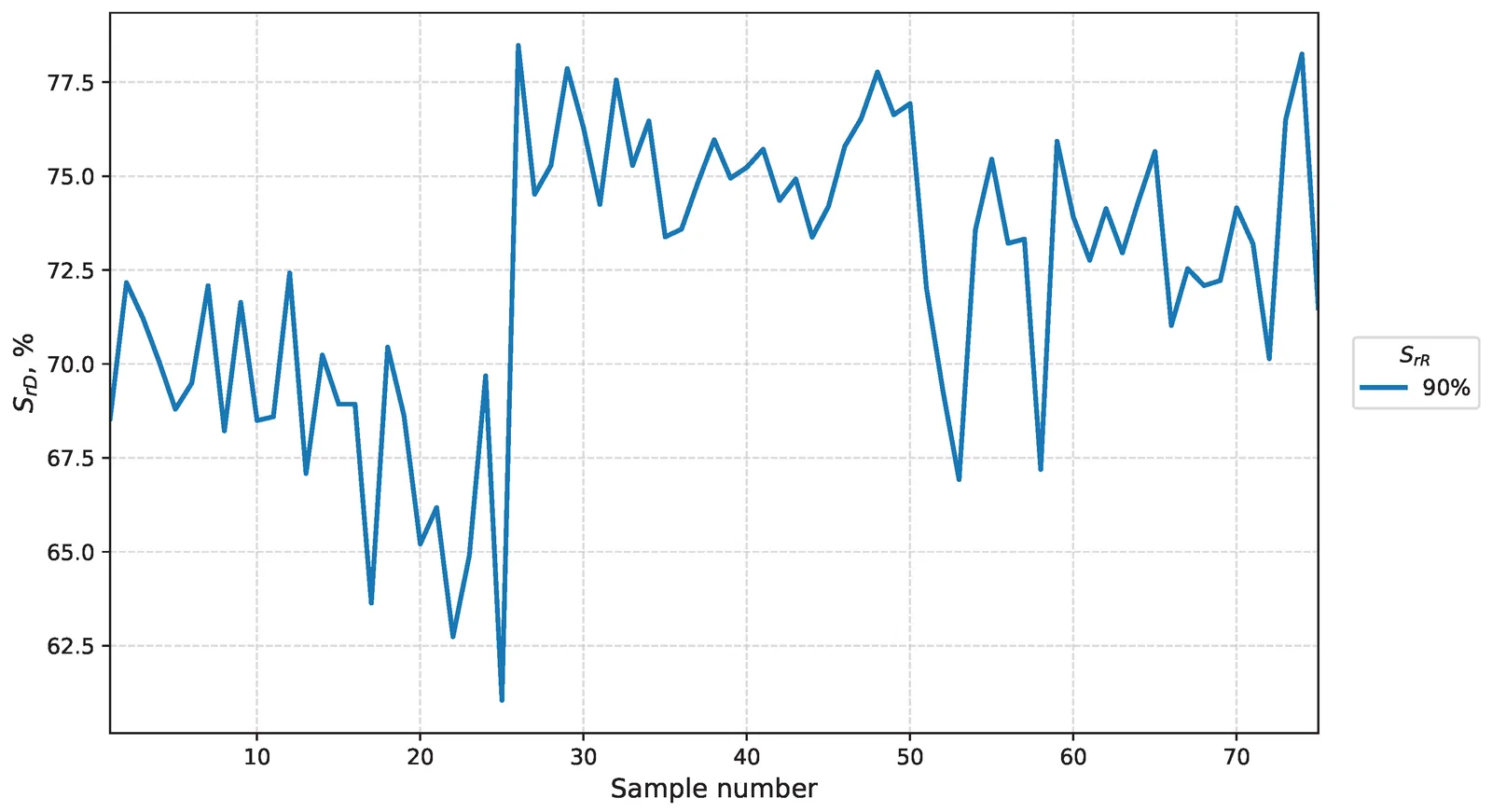
Calculated moisture content obtained from hyperspectral signal analysis for sand samples with moisture contents of 90%.

Next, the moisture coefficient $W_{r}$ was calculated from the filtered spectral radiance $L_{F}$ according to Equation (22) for the spectral ranges $\Delta \lambda_{A}$ (for λ∈〈1200,1250〉) and $\Delta \lambda_{B}$ (for λ∈〈1420,1460〉). Representative values of the moisture coefficient $W_{r}$, together with the fitted approximation function, are presented in Figure 10. Based on the calculated values of $W_{r}$, the moisture contents of the investigated sand samples were determined. Figure 14 and Figure 15 show the estimated moisture contents for samples with reference moisture levels ranging from 10% to 90%. For reference moisture levels between 10% and 80%, the estimated values generally remain close to the corresponding reference levels, although fluctuations between individual samples are observed. The magnitude and variability of these deviations differ depending on the moisture level. For samples with a reference moisture content of 90%, the estimated values range approximately from 61% to 78%, remaining below the reference value throughout the dataset.

**Figure 16:**
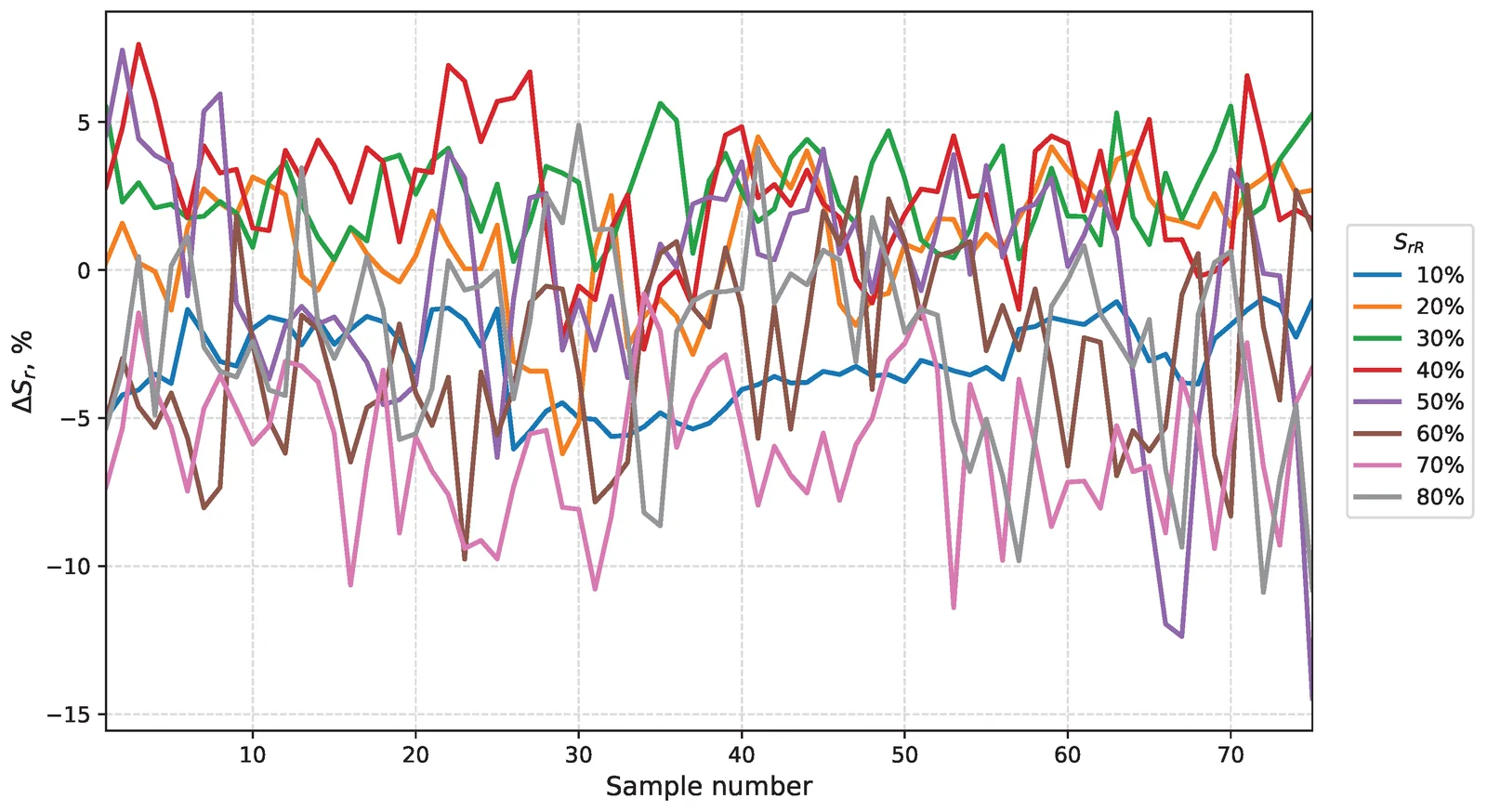
Absolute error of the proposed moisture estimation method for sand samples with moisture contents ranging from 10% to 80%.

**Figure 17:**
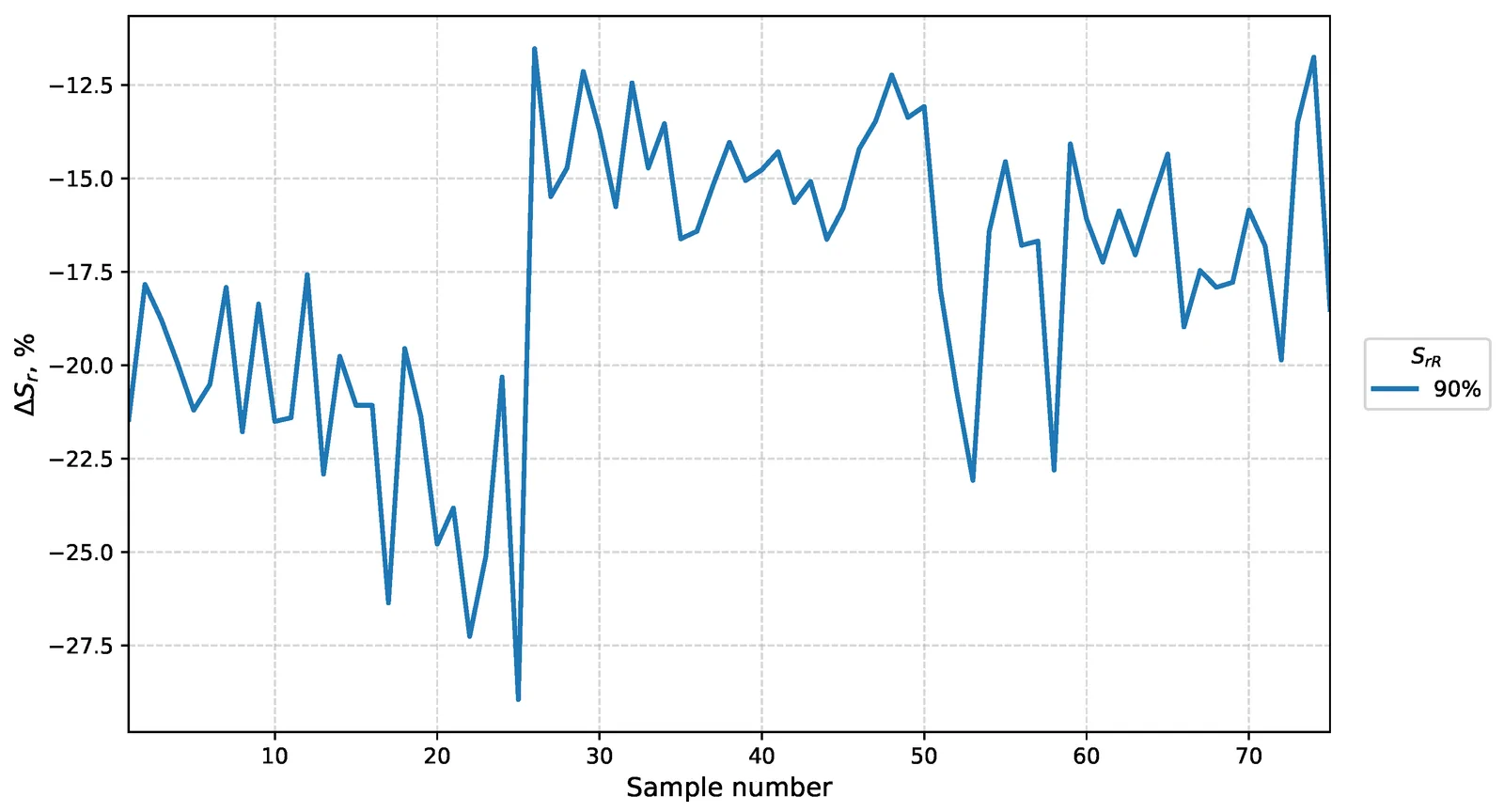
Absolute error of the proposed moisture estimation method for sand samples with moisture contents of 90%.

The absolute errors presented in Figure 16 and Figure 17 further illustrate these differences. For moisture levels between 10% and 80%, the errors fluctuate around zero, with varying magnitudes and distributions depending on the reference moisture content. In contrast, all errors for the 90% moisture level are negative, confirming the underestimation observed in Figure 15.

To provide a quantitative assessment of the estimation performance at individual saturation levels, the mean absolute error (MAE), root mean square error (RMSE), and mean bias error (MBE) were calculated. The results are summarized in Table 3.

**Figure 18:**
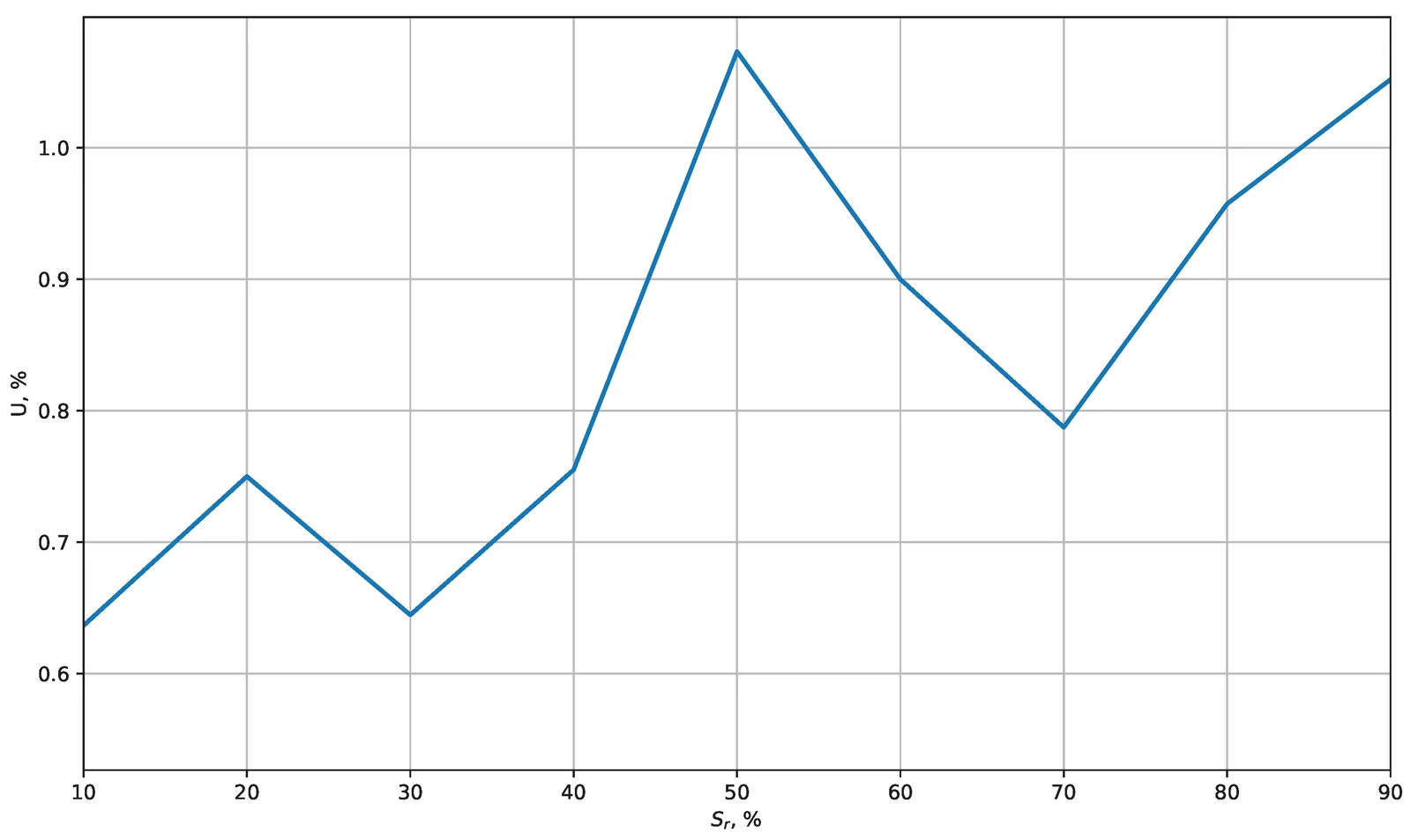
Expanded uncertainty of the proposed moisture estimation method for sand samples with different moisture contents.

Figure 18 presents the expanded uncertainty of the moisture estimation method as a function of the reference moisture content. The uncertainty ranges from approximately 0.55 to 1.07 percentage points across the investigated moisture levels. The lowest value occurs at 10% moisture, whereas the highest is observed at 50%. Additional increases are visible at 80% and 90% moisture.

The results indicate that the performance of the proposed method depends on the degree of saturation of the investigated sand samples. For reference sand samples with moisture contents ranging from 10% to 80%, the maximum absolute error did not exceed approximately 13.0%. Throughout the analyzed saturation range, including the 90% saturation samples, the expanded uncertainty at a 95% confidence level remained below 1.1%, demonstrating the potential of the proposed method for quantitative assessment of sand saturation under the tested laboratory conditions. However, for the 90% moisture samples, a systematic underestimation of the estimated moisture content was observed, resulting in absolute errors of up to approximately 29.0%.

Based on observations made during the measurements, the substantial underestimation observed at a saturation level of 90% is attributed to the presence of excess water at the sample surface. Due to the porous structure of sand, water initially occupies the pore spaces between grains. At high saturation levels, excess water can migrate toward the surface, forming a thin water layer. This layer changes the optical properties of the sample and affects the interaction of near-infrared radiation with the sand, thereby altering the recorded spectral response. Furthermore, at high moisture levels, the recorded radiometric signals become similar for different saturation levels, making it difficult to distinguish between them. Consequently, the relationship between the spectral coefficient and the degree of saturation established during calibration may no longer adequately describe the response of highly saturated sand, leading to the observed systematic underestimation.

## 7. Conclusions

As part of this study, a method, an algorithm, and a measurement system were developed to estimate the degree of saturation of sand using hyperspectral data acquired in the near-infrared (NIR) spectral range. The proposed method is based on exponential-function approximation and enables the estimation of sand saturation levels ranging from 10% to 80%, with maximum absolute errors of approximately 13 percentage points. However, at a saturation level of 90%, the estimation accuracy deteriorates substantially. Based on observations made during the measurements, this increased error is attributed to the formation of a thin water layer across the sample surface, which modifies its optical properties and limits the accuracy of the proposed model. The method is based on a radiometric model in which the spectral range recorded by the hyperspectral camera is divided into two subranges. The first subrange exhibits a strong dependence of the recorded signal on the degree of saturation, whereas the second shows substantially lower sensitivity to changes in saturation.

The radiance-based moisture model exhibited close agreement with the experimental data. However, radiance is strongly dependent on measurement conditions, including illumination intensity, measurement geometry, and the characteristics of the imaging sensor. In practical applications, these factors may affect the stability of the model and limit its applicability under varying environmental conditions. Therefore, a ratio-based moisture coefficient was introduced to reduce the influence of variations in incident radiation.

The effectiveness of the proposed moisture measurement method was validated by performing moisture measurements on sand samples with precisely controlled moisture contents. The obtained results demonstrate that the proposed method enables the practical implementation of a hyperspectral measurement system capable of accurate and rapid remote moisture estimation.

The obtained results are consistent with the relationships reported in the literature concerning the influence of moisture on the spectral characteristics of porous materials. Similar to the studies by Lobell and Asner [24] and Sadeghi et al. [33], a decrease in radiance with increasing moisture content and an exponential relationship between the spectral signal and water content were observed. These findings also highlight the importance of selecting appropriate spectral regions for moisture estimation, as the sensitivity of the measured signal to water content varies with wavelength. However, differences in soil properties and measurement conditions should be considered when transferring calibration models to materials other than those investigated in the original experiments.

Machine learning approaches, such as Random Forest and multilayer perceptron (MLP) neural networks, have also been proposed for soil moisture estimation. Lim et al. [34] achieved high moisture estimation accuracy using hyperspectral data and the corresponding reflectance derived from these measurements. The model developed in this study, based on radiance measurements and the ratio-based moisture coefficient derived from them, which is insensitive to changes in illumination conditions, is considerably simpler than machine learning methods. Consequently, it provides a more straightforward interpretation of the underlying physical relationships while reducing the computational complexity of the model. This makes the proposed approach particularly suitable for real-time processing of hyperspectral data acquired over large geographical areas. The conducted study demonstrated the feasibility of using near-infrared hyperspectral imaging to estimate the degree of saturation of sand under controlled laboratory conditions. The developed model and dedicated software enabled non-contact estimation based on the spectral characteristics of the investigated samples. However, the measurements were conducted under simplified laboratory conditions and did not systematically account for the variability of soil properties and environmental factors that may affect the recorded spectral response. In particular, differences in mineralogical composition, grain size distribution, particle shape, sand layer thickness, and surface roughness may influence light scattering and the measured signal. Furthermore, non-uniform moisture distribution may cause the recorded spectrum to represent primarily the near-surface region rather than the average degree of saturation of the entire sample. Consequently, the applicability of the proposed method beyond the laboratory setup has not yet been established.

Further research should investigate the influence of these factors through experiments involving different sand types, layer thicknesses, surface conditions, and moisture distributions. Validation using independently acquired hyperspectral data and measurements under varying illumination and environmental conditions will also be necessary to assess the robustness and generalizability of the method. These investigations will help determine the suitability of the proposed approach for practical applications, including environmental monitoring, precision agriculture, and airborne or satellite remote sensing.

## Figures and Tables

**Table 1:** Technical specifications of the Specim FX17 hyperspectral camera manufactured by SPECIM.

Parameter	Value
Spectral range	900–1700 nm
Spectral bands	224
Spectral resolution (FWHM)	8 nm
Spectral sampling/pixel	3.5 nm
Frame Rate	527
Lens FOV	40°
Aperture	F/1.7
Sensor material	InGaAs
Dimensions	170 × 85 × 85 mm
Operating temperature	+5–+40 °C

**Table 2:** Technical specifications of the LabScanner system.

Parameter	Value
Sample tray dimensions	400 mm × 200 mm
Max sample weight	5 kg
Scanning speed	0.1–99 mm/s
Control interface	RS232

**Table 3:** Error metrics for individual reference saturation levels.

Reference Saturation (%)	MAE (p.p.)	RMSE (p.p.)	MBE (p.p.)
10	3.10	3.39	−3.10
20	1.97	2.36	0.91
30	2.61	2.97	2.61
40	2.90	3.41	2.60
50	2.91	3.96	−0.23
60	3.48	4.18	−2.86
70	5.91	6.38	−5.91
80	3.06	4.09	−2.35
90	17.71	18.12	−17.71

## Data Availability

Data used in the study is available upon request.
